# Stepwise Evolution of *E. coli* C and ΦX174 Reveals Unexpected Lipopolysaccharide (LPS) Diversity

**DOI:** 10.1093/molbev/msad154

**Published:** 2023-07-03

**Authors:** Jordan Romeyer Dherbey, Lavisha Parab, Jenna Gallie, Frederic Bertels

**Affiliations:** Department Microbial Population Biology, Research Group Microbial Molecular Evolution, Max Planck Institute for Evolutionary Biology, Plön, Germany; Department Microbial Population Biology, Research Group Microbial Molecular Evolution, Max Planck Institute for Evolutionary Biology, Plön, Germany; Department of Theoretical Biology, Research Group Microbial Evolutionary Dynamics, Max Planck Institute for Evolutionary Biology, Plön, Germany; Department Microbial Population Biology, Research Group Microbial Molecular Evolution, Max Planck Institute for Evolutionary Biology, Plön, Germany

**Keywords:** LPS, overcoming host resistance, genotype–phenotype maps, experimental evolution, ΦX174, *E. coli* C

## Abstract

Phage therapy is a promising method for the treatment of multidrug-resistant bacterial infections. However, its long-term efficacy depends on understanding the evolutionary effects of the treatment. Current knowledge of such evolutionary effects is lacking, even in well-studied systems. We used the bacterium *Escherichia coli* C and its bacteriophage ΦX174, which infects cells using host lipopolysaccharide (LPS) molecules. We first generated 31 bacterial mutants resistant to ΦX174 infection. Based on the genes disrupted by these mutations, we predicted that these *E. coli* C mutants collectively produce eight unique LPS structures. We then developed a series of evolution experiments to select for ΦX174 mutants capable of infecting the resistant strains. During phage adaptation, we distinguished two types of phage resistance: one that was easily overcome by ΦX174 with few mutational steps (“easy” resistance) and one that was more difficult to overcome (“hard” resistance). We found that increasing the diversity of the host and phage populations could accelerate the adaptation of phage ΦX174 to overcome the hard resistance phenotype. From these experiments, we isolated 16 ΦX174 mutants that, together, can infect all 31 initially resistant *E. coli* C mutants. Upon determining the infectivity profiles of these 16 evolved phages, we uncovered 14 distinct profiles. Given that only eight profiles are anticipated if the LPS predictions are correct, our findings highlight that the current understanding of LPS biology is insufficient to accurately forecast the evolutionary outcomes of bacterial populations infected by phage.

## Introduction

Multidrug-resistant bacterial infections are one of the most pressing issues in medicine, a situation that is only expected to worsen in the coming decades (Centers for Disease Control and Prevention (U.S.) 2019; WHO 2021; Murray et al. 2022). An alternative to treating bacterial infections with antibiotics is phage therapy. Major research efforts are being conducted to develop efficient therapies (Oechslin 2018; Bull et al. 2019; Monteiro et al. 2019). An efficient therapy should be able to eliminate a pathogen without selecting for resistance to the phage being used to treat that pathogen. Phage resistance can often evolve by modifying the cell envelope to avoid phage adsorption. The outer membrane of all Gram-negative bacteria is covered in lipopolysaccharides (LPSs), which is consequently the first contact point between a bacterium and its environment (Zhang et al. 2013). LPS structure determines whether a bacterium will be recognized by the immune system, the efficiency of nutrient uptake as well as the susceptibility to antibiotics, and, importantly, the susceptibility to phage infection (Nikaido 2003; Simpson and Trent 2019; Mutalik et al. 2020). Hence, LPS structure is an important factor determining the success of phage therapy.

The functions of genes involved in synthesizing and assembling bacterial LPS have been elucidated (Schnaitman and Klena 1993; Amor et al. 2000; Raetz and Whitfield 2002; Qian et al. 2014; Klein and Raina 2019). Most *Escherichia coli* (*E. coli*) strains produce LPS molecules composed of three parts: the lipid A, the core oligosaccharide (OS), and the O-antigen chain. The LPS core OS itself consists of a very conserved inner core across *E. coli* species and a variable outer core (Amor et al. 2000). Strains with the tripartite LPS molecules (lipid A, core OS, and O-antigen) are termed “smooth” (Raetz and Whitfield 2002). Some *E. coli* strains, however, produce LPS molecules that lack one or more components. LPS molecules lacking the O-antigen are classified as “rough” and molecules lacking both the O-antigen and the outer-core OS as “deep rough” (van der Ley et al. 1986; Schnaitman and Klena 1993; Yethon et al. 1998; Whitfield et al. 1999; Amor et al. 2000; Klein et al. 2013). These modifications in LPS structure can lead to a plethora of phenotypic effects (Simpson and Trent 2019), including 1) destabilization of the outer membrane (Raetz and Whitfield 2002); 2) changes in the expression of some outer membrane proteins; 3) modification of intracellular turgor pressure (Pagnout et al. 2019); 4) increased susceptibility to hydrophobic compounds such as antimicrobial peptides, antibiotics, and bacteriocins (van der Ley et al. 1986; Schnaitman and Klena 1993; Yethon et al. 1998; Whitfield et al. 1999; Amor et al. 2000; Klein et al. 2013); 5) altered interactions with the host immune system (Raetz and Whitfield 2002; Matsuura 2013; Maldonado et al. 2016); 6) alteration of the redox status of cells leading to oxidative stress (Seregina et al. 2022); and 7) changes in resistance to phages (Hancock and Reeves 1976; Labrie et al. 2010; Kulikov et al. 2019; Mutalik et al. 2020).

**Fig. 1.**
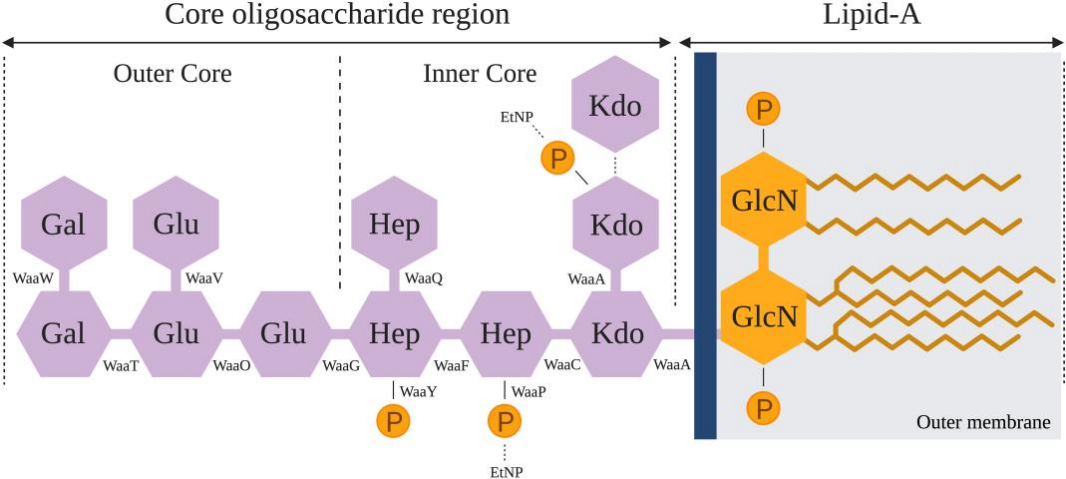
Structure of *E. coli* C's rough type LPS. The rough type LPS of *E. coli* C is composed of two parts: lipid A (composed of an acetylated and 1,4′-diphosporylated β(1→6)-linked glucosamine (GlcN) disaccharide) and the core OS (Raetz and Whitfield 2002). The core OS is subdivided into a structurally conserved inner core and an outer core. The dashed lines show nonstoichiometric substitutions of phosphate (P), ethanolamine (EtNP), and 3-deoxy-d-*manno*-octulosonic acid (Kdo) residues on the LPS (Kojima et al. 2010). Details of the LPS assembly process are provided in supplementary figure S1, Supplementary Material online.

Phage ΦX174 is a small (∼320 Å), tailless, lytic coliphage from the *Microviridae* family. It carries a circular, single-stranded DNA genome of only 5,386 bases long that encodes 11 genes (Sinsheimer 1959; Sanger et al. 1978). To enter a host cell, ΦX174 relies solely on attaching to the core OS by one of its spike proteins before migrating and irreversibly adsorbing to the host's cell surface via its capsid (Incardona and Selvidge 1973; Incardona et al. 1985; Sun et al. 2017). ΦX174 does it with a high degree of specificity. For instance, among 783 different *E. coli* isolates, only six (0.8%) could be infected by wildtype (WT) ΦX174 (Michel et al. 2010). In the laboratory, ΦX174 infects—and hence is usually grown on—*E. coli* C, which produces rough type (i.e., lacking in O-antigen) LPS molecules (Feige and Stirm 1976; Inagaki et al. 2000; Kawaura et al. 2000; Król et al. 2019) (fig. 1 and supplementary fig. S1).

Here, we explore the evolutionary potential of *E. coli* C to become resistant to infection by ΦX174 and, conversely, the potential of ΦX174 mutants to infect phage-resistant *E. coli* C hosts with modified LPS structures. We first generated a set of 31 *E. coli* C mutants that are resistant to infection by ΦX174 WT and determined that each mutant carries at least one mutation affecting LPS biosynthesis.

The currently accepted model for predicting LPS structure posits that the presence or absence of each LPS gene leads to a single LPS phenotype. Based on the position of each mutation, the one-gene-one-phenotype model allows us to predict the LPS structure of each of our LPS mutants. Our 31 *E. coli* C mutants collectively produce LPS molecules with eight distinct structures. To test the accuracy of these predictions, we evolved and genotypically characterized a set of 16 ΦX174 genotypes that can, together, infect all initially resistant 31 *E. coli* C mutants. If the LPS structure predictions are correct, then all bacteria predicted to carry the same LPS structure should be susceptible to the same set of phages. Interestingly, this was not always the case, indicating that the diversity of LPS structures generated by LPS-pathway mutations is much higher than anticipated from previous studies. Bacterial strains with different mutations in the same LPS gene sometimes have strikingly different infection phenotypes. Even the required number of phage mutations to overcome these resistances can differ significantly. A better understanding of the evolution of phage resistance hence requires a better understanding of the biology and evolution of LPS structures.

## Results

### Diverse LPS Structures Confer Resistance to ΦX174 Infection

To study LPS diversity in *E. coli* C, we generated 35 spontaneous phage-resistant strains and used whole-genome resequencing to identify the mutations conferring resistance (see Materials and Methods). Due to a lack of isogeneity or only partial resistance to ΦX174 WT, four of these strains (*E. coli* C R1, R3, R15, and R19) were excluded from downstream analyses (see supplementary text S1, Supplementary Material online). Of the remaining 31 strains, 27 are predicted to carry a single mutation, three carry two mutations, and one carries three mutations (see supplementary table S1, Supplementary Material online). A total of 36 mutations were identified, 32 of which are unique (including 15 nucleotide substitutions, 13 deletions, one duplication, and three IS*4* and IS*5* insertion events). Twenty-four (of 32, or 75%) mutations introduce premature stop codons or lead to frameshifts and thus are highly likely to disrupt gene function.

Interestingly, two mutations are shared by two strains (R27/R31 and R8/R10), and one mutation is shared by three strains (R4/R24/R29). Given that there are almost limitless ways to disrupt gene function, this high degree of parallelism is initially surprising. We note that the repetition of these mutations is unlikely to result from their positive selection. That is, phage-resistant *E. coli* C mutants of both low and high fitness are expected to have an equal chance of appearing on our agar plates as long as they can form visible colonies (Luria and Delbrück 1943). Presumably, the observed parallelism is, instead, due to elevated mutation rates at particular genomic positions (Lind et al. 2019). The fact that two of three parallel mutations occur in homopolymeric tracts supports this hypothesis (Moxon et al. 1994).

**Fig. 2.**
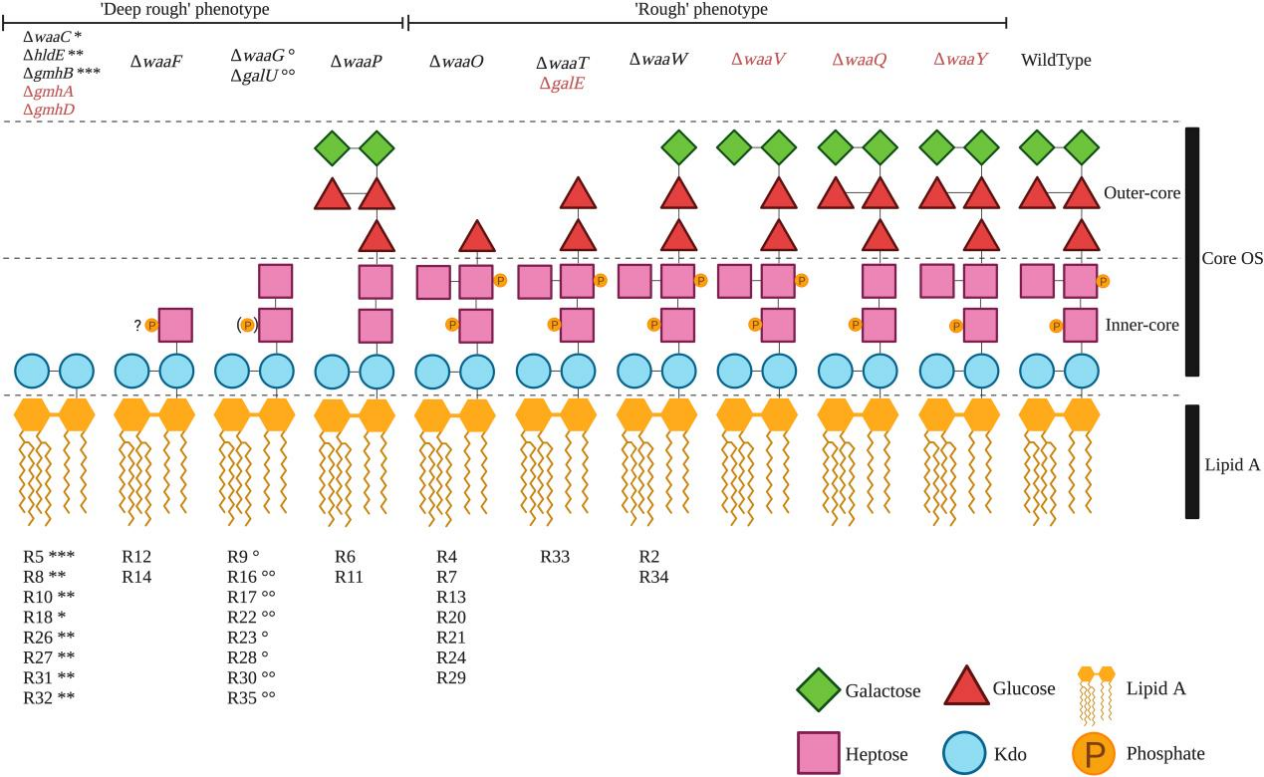
Predicted LPS structures of 30 ΦX174-resistant *E. coli* C strains. Predicted LPS structures fall into two groups depending on their degree of truncation and/or loss of phosphate groups: the “deep rough” phenotype with a completely truncated outer core or a loss of phosphate groups (20 *E. coli* C mutants) and the “rough” phenotype with smaller LPS truncations located in the outer core (ten *E. coli* C mutants). The LPS phenotype of the final *E. coli* C mutant (R25) could not be predicted (see text). The products of genes in red are also involved in LPS biosynthesis and, although no mutations were identified in these genes during this study, are also potential targets for phage resistance. Details on the functions of *gmhA*, *gmhB*, *gmhD*, *hldE*, *galU*, and *galE* are provided in supplementary figure S2, Supplementary Material online. “?P”: No information was found on the phosphorylation of Hep(I) in the absence of *waaF*; (P): only 40% of the hexose phosphorylation was observed (Yethon et al. 2000); R#: the number gave to each *E. coli* C resistant strain; and “*, **, ***, °, °°”: symbols link the resistant strains (R#) to particular mutated LPS genes (Δ*gene-names*). Resistant strains with identical symbols carry their mutations in the same LPS gene. For example, R8, R10, R26, R27, R31, and R32 strains each carry a mutation in the *hldE* gene, and R9, R23, and R28 strains all carry a mutation in the *waaG* gene (supplementary table S1, Supplementary Material online).

Mutations in genes encoding the LPS machinery are expected to lead to changes in LPS structure and, in some cases, a switch from a rough to a deep rough phenotype (Yethon et al. 1998; Whitfield et al. 1999; Yethon et al. 2000). It has been shown that logical predictions of mutant LPS structures can be made. If an enzyme that synthesizes a particular LPS component is absent or nonfunctional, then that LPS component cannot be synthesized. In addition, LPS components assembled downstream of the missing component are also absent from the final LPS structure. The assumption that each mutation in an LPS gene leads to a complete lack of function allows us to predict the LPS structure and type of each of the 31 *E. coli* C phage-resistant mutants (fig. 2 and supplementary figs. S1 and S2 and table S1, Supplementary Material online). A total of seven emergent LPS structures were predicted from our set of mutants (fig. 2). Compared with the *E. coli* C WT LPS, three of these seven LPS structures carry alterations in the outer-core LPS and hence are predicted to be of the rough type (as is *E. coli* C WT; ten mutants). The remaining four LPS structures differ from WT LPS in both the inner- and outer-core LPS and are presumed to be of the deep rough type (20 mutants) (see supplementary table S1, Supplementary Material online).

The LPS structure and type of the remaining mutant (*E. coli* C R25) were not predicted because the effect of the phage resistance mutation on LPS biosynthesis remains unclear. R25 carries a nonsense mutation in *rfaH*. The *rfaH* gene encodes a transcriptional antiterminator that regulates the *waa* operon (Bailey et al. 1997; Klein and Raina 2019), meaning that any number of LPS biosynthetic genes could potentially be affected by this mutation.

The LPS structure predictions above can be tested by evolving ΦX174 WT to specifically infect *E. coli* C cells with modified LPS structures. That is, a phage that can infect a given modified LPS structure is expected to be able to infect all bacterial strains displaying this LPS type, regardless of the underlying mutations. Alternatively, the inability of mutant phages to cross-infect bacterial strains of the same predicted LPS structural class (but via different mutations) would indicate that these mutations actually lead to distinct LPS structures.

### ΦX174 Evolves to Overcome Host Resistance: A Process That Can Be Accelerated by Host Diversity

**Fig. 3.**
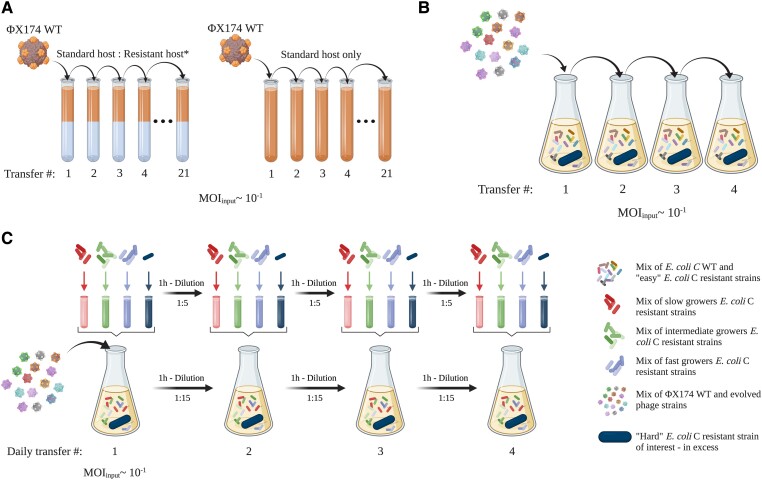
Three phage evolution experiments used to overcome *E. coli* C resistance to WT ΦX174. (*A*) First evolution experiment (standard): 31 independent lineages, each founded by ΦX174 WT, were serially transferred daily for up to 21 days (21 transfers). Phages were grown on nonevolving (i.e., freshly prepared at each transfer) host cultures containing a mixture of *E. coli* C WT and one of the 31 initially resistant strains (see supplementary table S1, Supplementary Material online). The presence of *E. coli* C WT was necessary to propagate ΦX174 and avoid its dilution from one transfer to another. Phages capable of infecting the initially resistant host strain were isolated from 12 lineages. Three control lineages were included, where ΦX174 was cultured on only *E. coli* C WT. (*B*) Second evolution experiment (increased diversity). A cocktail of ΦX174 WT and 14 evolved ΦX174 strains was generated (panel *A*—the two evolved phages infecting R5 and R19 were part of the final cocktail but removed from the mutational analysis, see supplementary text S1, Supplementary Material online). The phage cocktail was transferred daily for up to four days, on nonevolving host cultures containing 1) *E. coli* C WT, 2) the 14 host strains for which resistance had been overcome (permissive hosts), and 3) an excess of one of the still-resistant strain of interest (nonpermissive host). Phages capable of infecting two resistant strains of interest (*galU* and *waaG* mutants) were isolated from two lines. (*C*) Third evolution experiment (increased diversity and generations). The same cocktail used for the second evolution experiment (panel *B*) was transferred four times daily for four days (i.e., a maximum of 16 transfers) on nonevolving host cultures containing: 1) *E. coli* C WT, 2) the 14 strains for which resistance had been overcome (permissive hosts), and 3) one of the still-resistant strain of interest (nonpermissive host). Notably, all transfers completed on the same day involved transferring both phage and bacteria; supernatants were collected on every fourth transfer, and only phages were transferred. Phages capable of infecting two resistant strains of interest (*waaP/pssA* mutants) were isolated from two lines. Details of the evolved phages are presented in supplementary table S2, Supplementary Material online, and further methodological details are provided in Materials and Methods.

Phages that can infect resistant *E. coli* C mutants were evolved by serially transferring ΦX174 WT on each of the 31 resistant *E. coli* C strains (fig. 3*A* and Materials and Methods). To reduce the complexity of the experiment, bacterial cultures were not allowed to coevolve. Instead, phages were inoculated into fresh, exponentially growing host cultures at each transfer. The experiment gave rise to 12 evolved phages that were collectively able to infect 21 (of the 31) initially resistant *E. coli* C strains (these display the “easy” resistant phenotype; see supplementary table S1, Supplementary Material online). However, even after 21 transfers, ten (of 31) resistant *E. coli* C strains remained uninfected (the “hard” resistant phenotype; see supplementary table S1, Supplementary Material online).

Overcoming phage resistance of this subset of hard-resistant strains may not be achievable with few mutational steps. Hence, either the evolution experiment has to be performed for a much longer period of time or an experiment that increases the genetic variation available to evolution has to be designed (Hermisson and Pennings 2005; Barrett and Schluter 2008). We designed two further evolution experiments, one to increase genetic diversity and another one to additionally increase the speed of evolution by increasing the number of phage generations per day (figs. 3*B*and 3*C*). In both of these experiments, phage diversity was increased by combining all evolved phage strains that successfully reinfected their corresponding resistant strains into a single phage cocktail (Hermisson and Pennings 2005; Barrett and Schluter 2008). The phage cocktail was serially transferred once per day in the overall second evolution experiment, whereas four transfers per day were performed in the third evolution experiment. Phage diversity was maintained from one transfer to another by adding all corresponding resistant strains to the host culture (see Materials and Methods). At the end of the evolution experiments, all resistant strains could be cross-infected by at least one of the evolved phage mutants. Our method of evolving phages with an extended infection range is simple and effective and allows the successful evolution and retrieval of phages that, together, can infect all of the 31 resistant *E. coli* C phenotypes.

**Fig. 4.**
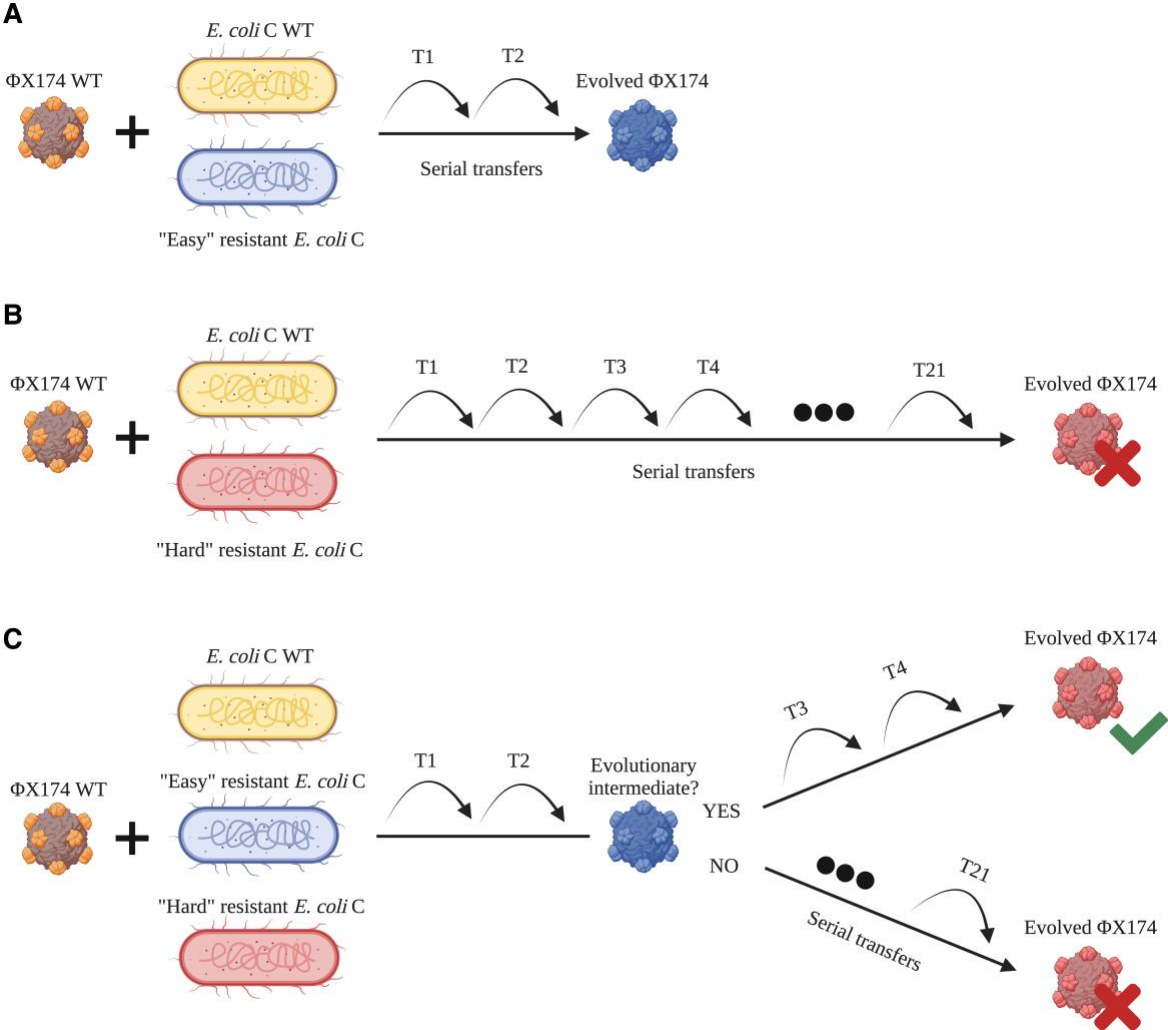
Increased host diversity facilitates viral adaptation. (*A*) ΦX174 WT evolves on *E. coli* C WT and an easy-resistant *E. coli* C strain. Adaptation to the resistant mutant is fast and only requires a few transfers. (*B*) ΦX174 WT evolves on *E. coli* C WT and a hard-resistant *E. coli* C strain that it failed to infect within 21 standard serial transfers. Infecting this resistant strain may be difficult and take a long time. (*C*) ΦX174 WT evolves in a three-host mix containing *E. coli* C WT and two *E. coli* C resistant strains: one easy and one hard. Phage adapts quickly to the easy-resistant phenotype. If the infection of the easy-resistant strain leads to the production of an evolutionary intermediate phage, then the hard phenotype can be infected after a few more transfers. If no evolutionary intermediate phage is produced, then no phage evolves to infect the hard-resistant *E. coli* C strain.

Increasing both phage and host diversities in the second and third evolution experiments accelerated the adaptation of ΦX174 to resistant *E. coli* C strains. Although our experiment quickly gave rise to phages able to infect most nonpermissive strains (fig. 4*A*), some phage-resistant hosts could only be infected when both host and phage diversities were increased (figs. 4*B*and 4*C*).

To identify the mutation(s) responsible for the reinfection of the resistant mutants, we performed whole-genome resequencing on the 16 evolved phages that successfully overcame the corresponding resistance in one of the three evolution experiments (see Materials and Methods). We found mutations in *F* and/or *H* genes in all 16 evolved phages (supplementary table S2, Supplementary Material online). Notably, all hard-resistant *E. coli* C strains—those whose resistance was overcome in the second and/or third evolution experiments—are only infected by phages carrying four mutations (see supplementary tables S1 and S2, Supplementary Material online).

The evolutionary emergence of a multimutation phage in a two-strain evolution experiment is expected to require a large number of transfers (fig. 4*B*). However, this process is significantly accelerated by propagating a diverse mixture of phages. If some of the phages are evolutionary intermediates (i.e., carry a subset of mutations required to overcome hard resistance), then the mutations required to overcome hard resistance can emerge in a single mutational step, either by recombination (Rokyta et al. 2006) or by adding mutation to an existing set (fig. 4*C*). For example, the hard-resistant strain *E. coli* C R22 (a *galU* mutant; see supplementary table S1, Supplementary Material online) is overcome in the second evolution experiment by ΦX174 R22 T1, a phage that carries four mutations (three in *F*, one in *H*; see supplementary table S2, Supplementary Material online). Each of these mutations was present in the phage cocktail used to initiate the evolutionary lineage: ΦX174 R18 T4 and ΦX174 R8 T6 contain three of the four mutations, and the final mutation is found in ΦX174 R10 T6. The evolved phage infecting R22 may, therefore, be the product of a recombination event between ΦX174 R10 T6 and ΦX174 R18 T4 (or ΦX174 R8 T6). Alternatively, ΦX174 R18 T4 or ΦX174 R8 T6 could have acquired a single additional mutation. Thus, adaptation to certain easy-resistant LPS phenotypes yields evolutionary intermediate phages that can recombine or acquire a few additional mutations to infect a hard-resistant LPS structure.

Our results show that high host diversity can facilitate the evolution of phages to infect resistant strains. In contrast, Sant et al. showed that high host diversity hinders phage evolution (Sant et al. 2021). The major difference between the experiments is the difficulty of the evolutionary problem posed. Sant et al. only evolved phages to infect easy-resistant strains, strains that could be infected by phages after seven to eight transfers through the acquisition of a few mutations. Evolutionary intermediates will not be required in this situation (fig. 4*A*). However, as we show here, it is very difficult to infect hard-resistant strains starting with low phage and host diversities (fig. 4*B*). Hence, increasing the host diversity will accelerate the evolution of phage adaptation under conditions where the additional hosts allow the evolution of intermediate phage phenotypes (fig. 4*C*).

### ΦX174 Overcomes *E. coli* C Resistance by Mutations in the F Capsid and H Minor Spike Proteins

**Fig. 5.**
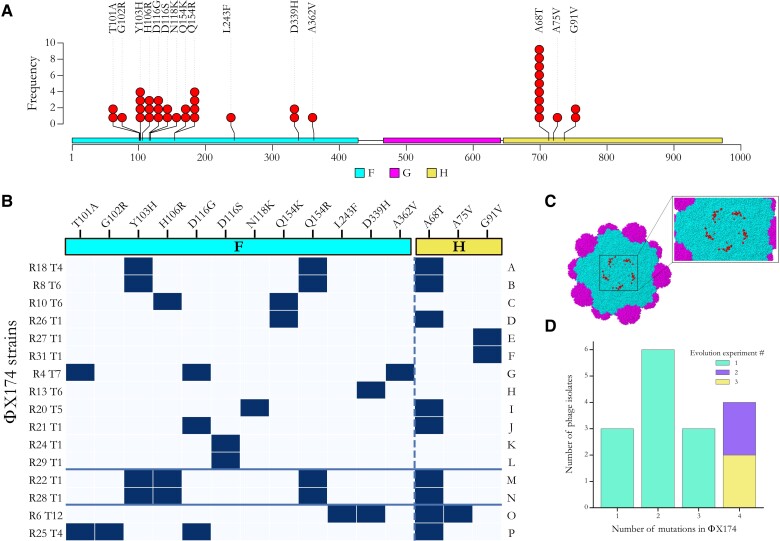
Adaptation of ΦX174 to resistant strains of *E. coli* C. (*A*) Positions of all predicted amino acid changes in the F (viral capsid; colored in cyan) and H (minor spike; colored in yellow) proteins observed in the 16 evolved ΦX174 strains. No mutation was found in the G protein (major spike; colored in magenta) despite being an integral part of ΦX174's recognition system and the first contact point with the LPS structure (Kawaura et al. 2000; Sun et al. 2017). (*B*) Substitution matrix of all amino acid changes observed in ΦX174. ΦX174 strains are ordered based on the predicted LPS structures they infected during the evolution experiments (A–F, overcame heptoseless *waaC* and *hldE* mutants; G–L, overcame *waaO* mutants; M–N, overcame *galU* and *waaG* mutants; O, overcame *waaP*/*pssA* mutant; and P, overcame *rfaH* mutant). The solid blue lines separate the evolved phages according to the evolution experiment from which they were isolated (A–L, first; M–N, second; and O–P, third). The x-axis shows amino acid substitutions in the F and H proteins (separated by the dashed line), R# indicates the resistant bacterial strain the phage evolved on, and T# is the number of transfers required to overcome host resistance. Amino acid numbering in the *F* gene starts from the initial Methionine and may differ by one to numberings in publications where the Methionine is subtracted. (*C*) Location of protein changes on the ΦX174 three-dimensional structure. The F capsid proteins are colored in cyan, and the G major spike proteins are in magenta. Residues affected by the mutations in this study are in red and shown for one F capsid unit only. The G protein spike (above that pictured here) has been removed to improve visualization. The image was generated using Geneious Prime (version 2020.1.2) and Protein Data Bank accession code 2BPA (McKenna et al. 1992). Changes in the H protein cannot be displayed as they all occur outside of the currently determined crystallographic structure. (*D*) Barplot of the number of evolved phage isolates carrying one, two, three, and four mutations. Most phage isolates—particularly those isolated from the second and third evolution experiments—carry multiple mutations.

The 16 evolved phages collectively carry 40 mutations, 15 of which are unique (fig. 5*A* and supplementary table S2, Supplementary Material online). All are nonsynonymous and occur in either *F* (encoding the viral capsid) or *H* (encoding the minor spike protein involved in viral DNA injection) genes (figs. 5*B*and 5*C*). The majority of phages (13 of 16) carry between two and four mutations (fig. 5*D*). These results are consistent with previous studies demonstrating that mutations in *F* and *H* are relevant for the adaptation of ΦX174 to novel hosts (Weisbeek et al. 1973; Crill et al. 2000; Cox and Putonti 2010).

Parallel evolution was common in our evolved phages, particularly in the control lines and the lineages that failed to infect their corresponding resistant *E. coli* C strains during the first evolution experiment (see supplementary table S3, Supplementary Material online). Of the three controls and 15 resistant lines that remained uninfected by their own phage, a total of 33 mutations were detected, but only ten were unique. All these mutations are located in the *F* gene, except one in the A/A* genes. *A* gene is involved in the stages II and III of phage DNA replication (Barrell et al. 1976; Sanger et al. 1978), whereas the A* gene is a nonessential gene that may play a role in the inhibition of host cell DNA replication and superinfection exclusion (Eisenberg and Ascarelli 1981). The mutation in the *F* gene at genomic position 2280 (causing amino acid change S427L in the F protein) arose in 13 lineages. Among them, nine (69%) carry between one and three additional mutations. High degrees of parallel evolution have also previously been observed in ΦX174 WT strains and other viruses (Bull et al. 1997; Wichman et al. 1999; Remold et al. 2008; Bertels et al. 2019; Bertels et al. 2021) and are an indicator of either 1) a low number of mutational targets with similar high fitness gains (many more targets with lower fitness may exist, but these will not be outcompeted by the high fitness mutants) or 2) mutational bias toward certain positions in the genome (Lind et al. 2019; Bertels et al. 2021). Although these substitutions may not allow the infection of resistant *E. coli* C strains, they are probably adaptations to our evolution experiment.

We also observed high levels of parallel evolution in the phage strains (isolates) that evolved to infect *E. coli* C mutants with the same predicted LPS phenotype (figs. 5*A*and 5*B*). Parallelism was especially remarkable among phages infecting the four bacterial strains with the shortest (deep rough heptoseless) predicted LPS structure (see fig. 2). The four phages infecting *E. coli* C R18, R8, R10, and R26 all carry an amino acid substitution at position 154 in the F protein (either Q154K or Q154R). In addition, the two phage strains adapted to the *E. coli* C mutants R8 (*hldE* mutant) and R18 (*waaC* mutant) also carry the mutations Y103H in the F protein and A68T in the H protein. Finally, whereas *E. coli* C R27 and R31 both became resistant via the same amino acid substitution in HldE (G27A), their corresponding phages evolved similarly by each acquiring a different mutation that led to the same change at the protein level (G91V in the H protein).

Parallel evolution was also observed in phages infecting bacteria within several other classes of predicted LPS structure. For example, the mutation at position 116 in the F protein (either D116G or D116S) occurred in four different phages all infecting different *waaO* mutants (figs. 5*A*and 5*B* and supplementary table S2, Supplementary Material online). Parallel evolution in phages that evolved to infect *E. coli* C strains with the same predicted LPS structure generally supports the predicted classes of LPS structure. However, analyses in the following section demonstrate that LPS structure predictions generally underestimate LPS diversity.

### Evolved Phages Can Be Used to Discriminate between LPS Phenotypes

To determine precisely which of the 16 evolved phages can infect which of the 31 initially resistant *E. coli* C strains each (i.e., the infection pattern of each phage), a cross-infection assay was performed (supplementary fig. S3, Supplementary Material online). This involved measuring the infectivity of all possible host–phage combinations by two distinct methods: 1) phage spotting on host lawns and 2) host spotting on phage lawns. In cases where a mismatch between the two results was observed, standard plaque assays were additionally applied (see Materials and Methods).

If our predictions—based on the current understanding in the literature—of seven distinct LPS structures among the 31 initially resistant *E. coli* C strains are accurate (see fig. 2 and supplementary table S1, Supplementary Material online), then all phages that evolved to infect a mutant with a given predicted LPS structure are expected to cross-infect all other host strains with the same predicted LPS structure. That is, the infection patterns in supplementary figure S3, Supplementary Material online would be identical for each class (color) of *E. coli* C mutant.

**Fig. 6.**
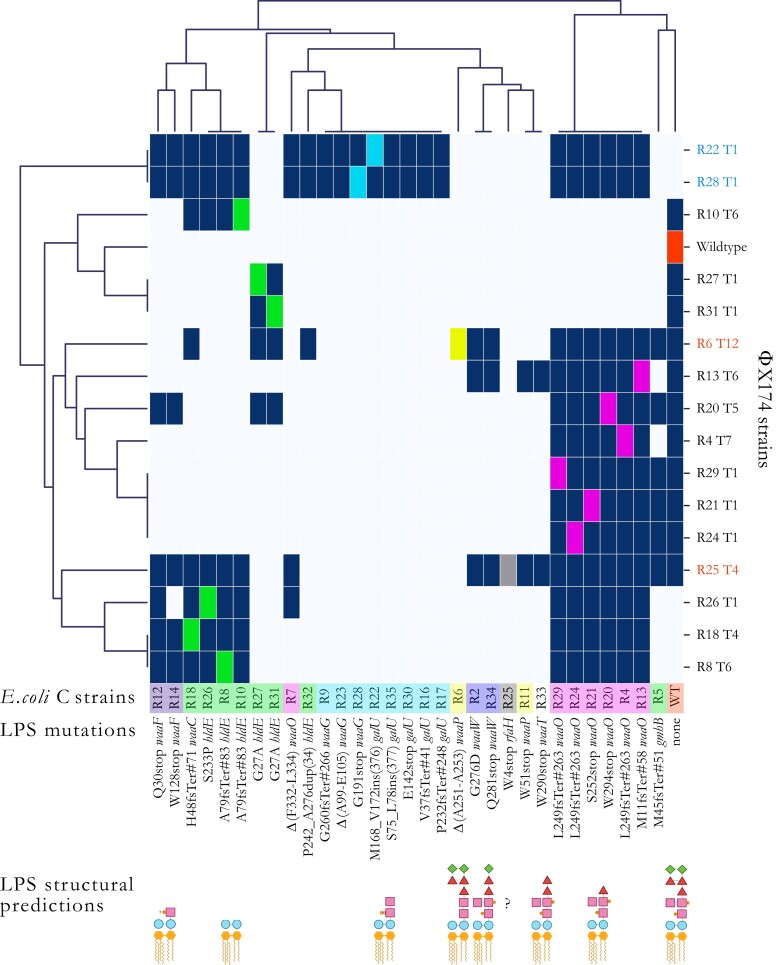
Hierarchical agglomerative clustering analysis of the host ranges of evolved phage. To unravel structures in the heatmap infection matrix (see supplementary fig. S3, Supplementary Material online), we performed a clustering analysis using the default settings of the Seaborn library (version 0.11.2) for Python (version 3.7.4). Briefly, each data point corresponds to either presence (dark blue) or absence (light blue) of phage infections determined from the combination of the different spotting test assays’ results (Materials and Methods). The linkage method computes the distance between them and then repeatedly combines the two nearest clusters into larger clusters until a single cluster is left. *Escherichia coli* C strains are colored based on their predicted core LPS structure (see fig. 2). Phage names in black were obtained during the first 21 serial transfers (first evolution experiment). Phage strains in blue were obtained in the second evolution experiment (phage cocktail, one transfer per day). Phage strains in red were obtained in the third evolution experiment (phage cocktail, four transfers per day). Dark blue squares, infection; light blue square, no infection; and colored square, control infection by a phage evolved on that host. R# indicates the number of the resistant strain the phage evolved on, and T# is the transfer number where plaques were first observed. “?”: core LPS structure of *E. coli* C R25 (*rfaH* mutant) could not be predicted. “INS”: insertion. “DUP”: duplication. stop, stop codon. “Δ”: deletion. Examples: L249fsTer#263 indicates a frameshift (fs) leading to a premature codon stop (Ter); the position of the premature stop codon is in parentheses. S75_L78ins(377) indicates an insertion; the two flanking amino acids are separated by a “_” and followed by the number of inserted amino acids in parentheses. Δ(A99-E105) indicated deletion; two flanking amino acids are separated by a “-”. P242_A276dup(34) indicates a duplication; the two flanking amino acids are separated by a “_” and followed by the number of duplicated amino acids in parentheses.

To visually determine whether infection patterns cluster according to the predicted LPS structures, a hierarchical agglomerative clustering analysis was performed on the infection matrix in supplementary figure S3, Supplementary Material online. Of the six predicted LPS structures that occur in at least two *E. coli* C mutants, three form unbroken clusters with respect to infectivity patterns (cyan, light purple, and dark purple highlighting in fig. 6) and three do not (magenta, yellow, and green highlighting). Each group is discussed in more detail below.

#### LPS Predictions That Cluster by Infection Pattern

The identical ΦX174 mutants R22 T1 and R28 T1 that evolved to infect resistant strains with mutations in *galU* (*E. coli* C R22) and *waaG* (*E. coli* C R28) can cross-infect all eight *E. coli* C mutants predicted to exhibit the same LPS structure (*E. coli* C R9, R16, R17, R22, R23, R28, R30, and R35; highlighted in cyan in fig. 6 and supplementary fig. S3, Supplementary Material online). Furthermore, both *E. coli* C *waaW* mutants (R2 and R34; light purple) are cross-infected by the same three evolved phage genotypes (R6 T12, R13 T6, and R25 T4). Also, both *E. coli* C *waaF* mutants (R12 and R14; dark purple) are infected by a near-identical set of seven (R12) or six (R14) phages. In each of these cases, phage infectivity patterns appear to be a good indicator of host LPS phenotype.

#### LPS Predictions That Do Not Cluster by Infection Pattern

Six of the seven *E. coli* C *waaO* mutants (highlighted in magenta in fig. 6 and supplementary fig. S3, Supplementary Material online) can be cross-infected by the same set of 13 phages. The seventh *waaO* mutant (*E. coli* C R7) was the only *waaO* mutant for which the corresponding phage lineage did not evolve to infect the resistant mutant in the first evolution experiment. It can only be infected by the phage infecting R26 and phages that evolved in the second and third evolution experiments (fig. 6). Hence, it is likely that the LPS structure of R7 differs from that of the other *waaO* mutants. The difference could conceivably result from residual (or altered) WaaO function; *E. coli* C R7 carries an in-frame deletion of a 6 bp repeat in *waaO*, which alters two WaaO residues but leaves the remainder of the protein intact (see supplementary table S1, Supplementary Material online). Because R7 is close to the *galU/waaG* mutants (fig. 6), it may be possible that its LPS structure resembles the ones of the *galU/waaG* mutants. However, more detailed LPS studies are needed to elucidate the LPS structure of R7.

The infection patterns of the two *waaP* mutants *E. coli* C R6 and R11 (highlighted in yellow in fig. 6 and Supplementary fig. S3, Supplementary Material online) also suggest that, in contrast to the prediction, these strains possess different LPS structures. R6 is infected by a single phage that evolved directly on host R6 (phage R6 T12 from the third evolution experiment), whereas R11 is cross-infected by two different phages (phage R13 T6 from the first evolution experiment and phage R25 T4 from the third evolution experiment). *E. coli* C R6 was the last resistant strain to be infected by an evolved phage, and the evolved phage infecting R6 cannot cross-infect R11.

The R11 mutation introduces a premature codon stop at the beginning of the *waaP* gene (fig. 6 and supplementary table S1, Supplementary Material online). *WaaP* is special because it is not required for completing the backbone structure of the LPS (Yethon et al. 1998). The deletion of this gene should, therefore, lead to a unique LPS structure (see fig. 2). Instead, the cluster map analysis indicates that R11 displays an LPS phenotype comparable with the rough LPS phenotype of R33, a *waaT* mutant (fig. 6). These similarities may be caused by a polar effect of the R11 mutation in *waaP* on *waaT* downstream. This effect may have resulted in a decrease or an alteration of *waaT* activity leading to a truncation of the outer-core LPS similar to the one in R33.

The combination of phages that can infect each of the eight heptoseless *E. coli* C mutants predicted to have the shortest LPS structure (fig. 2; green highlighting in fig. 6 and supplementary fig. S3, Supplementary Material online) is remarkably different. These *E. coli* C strains cluster into four different phenotypic subgroups (fig. 6). The first subgroup consists of *E. coli* C R8, R10, R18, and R26. Each of these strains can be cross-infected by their corresponding evolved phages, regardless of their genotypes (a mixture of *waaC* or *hldE* mutants). However, none of the four evolved phages can cross-infect *E. coli* C R27 or R31 (*hldE* mutants). *E. coli* C R27 and R31 comprise the second subgroup. Both *E. coli* C R27 and R31 and their corresponding evolved phages acquired identical genotypes, and, not surprisingly, they also share phenotypes. *E. coli* C R5 (*gmhB* mutant) and *E. coli* C R32 (*hldE* mutant) fall into a third and a fourth subgroups. *E. coli* C R32 is the only heptoseless mutant for which the corresponding phage lineage failed to infect during the first evolution experiment. Only a very narrow but different set of evolved phages can infect R5 and R32 (fig. 6 and supplementary fig. S3, Supplementary Material online).

A total loss of function in *hldE* or *waaC* genes always leads to a complete truncation of the inner core LPS (supplementary fig. S2*A*, Supplementary Material online). Thus, the involvement of these two genes in the production of the deep rough phenotypes (highlighted in green in figs. 6 and supplementary fig. S3, Supplementary Material online) cannot explain the vast phenotypic diversity in LPS structures. For example, *E. coli* C R8, R10, R18, and R26 belong to the same phenotypic group despite having both *hldE* or *waaC* mutated, whereas R27, R31, and R32 (all *hldE* mutants) do not belong to this group (fig. 6 and supplementary table S1, Supplementary Material online). Instead, the location and type of mutation are more likely to cause the observed LPS structure diversity.

Interestingly, HldE is a bifunctional protein, where each function is performed by a different domain: HldE1 (from M1 to T318) and HldE2 (from M344 to G477) (Valvano et al. 2000; Kneidinger et al. 2002; Raetz and Whitfield 2002; McArthur et al. 2005). Each domain has the potential to affect the LPS structure, but all four unique *hldE* mutations occur within the first domain only, resulting in at least three different LPS phenotypes (fig. 6 and supplementary table S1, Supplementary Material online). These LPS phenotypes are caused by three different types of mutations (duplication, missense mutations, and frameshift) in different parts of the same domain. Yet, there is no obvious correlation between the LPS phenotypes and mutation positions and/or types. The phenotypic diversity produced by these mutations in a single domain of a single gene is surprising and highlights our extremely limited understanding of LPS biology.

The mutated *gmhB* gene in *E. coli* C R5 is involved in the production of yet another deep rough phenotype. Previous research demonstrated that the deletion of *gmhB* only causes a partial defect in the synthesis of the LPS core, resulting in the formation and coexisting of a heptoseless and a heptose-rich form of LPS molecules (Kneidinger et al. 2002). The *gmhB* mutation in R5 is an early frameshift leading to a premature stop codon. Hence, we assume that the effect is similar to what was found by Kneidinger et al., a mix of deep rough and WT LPS molecules on the outer bacterial cell membrane. This hypothesis is supported by the fact that R5 most closely clusters with the WT in our infection matrix.

### Linking Phage Genotype to Phenotype

Phage infectivity patterns (host ranges) can be used to construct phage genotype–phenotype maps. For example, every phage strain that loses the ability to infect *E. coli* C WT has a combination of two mutations: A68T in the H protein and Q154K/R in the F protein (figs. 5*B* and 6). In isolation, A68T confers the ability to infect *waaO* mutants and Q154K/R to infect (R8, R18, R10, and R26)-heptoseless mutants. The two mutations combined allow the phage to infect both heptoseless and *waaO* mutants but cannot infect the *E. coli* C WT (figs. 5*B* and 6). ΦX174 R25 T4 is the only phage that can infect all three genotypes: *waaO*, the (R8, R18, R10, and R26)-heptoseless mutants, and *E. coli* C WT. Presumably, because this phage does not contain the Q154K/R mutation and instead uses another set of mutations that allows R25 to infect (R8, R18, R10, and R26)-heptoseless mutants (supplementary table S2, Supplementary Material online).

## Discussion

Our experiments have shown that *E. coli* C can become resistant to ΦX174 infection through mutations in genes involved in the LPS biosynthesis or assembly. ΦX174 can, in turn, evolve to reinfect initially resistant *E. coli* C strains by acquiring mutations in genes involved in host recognition or DNA injection. Similar to ΦX174 WT, the evolved phages are highly host-specific and hence can be used to distinguish between bacteria with different LPS structures. Such high host specificity allows testing of the currently accepted LPS structure model, where LPS structures are determined by the presence and absence of LPS synthesis and assembly genes. Our data on phage infectivity show that LPS diversity is far greater than the current model suggests. Presumably, the modulation of LPS gene activity leads to a variety of LPS phenotypes. Our findings can potentially be applied to other phages that use LPS as a single or complementary receptor for host infection (Bertozzi Silva et al. 2016; Latino et al. 2016; Broeker and Barbirz 2017). The LPS is a major structural component of the outer membrane of Gram-negative bacteria (Zhang et al. 2013), and its biosynthesis and assembly are conserved among bacterial species.

Another major finding of our study is that increasing phage and host diversity allows the infection of hard-resistant phenotypes (see fig. 4*C*). This phenomenon—the successful infection of hard-resistant phenotypes—is similar to a receptor shift observed with phage λ (Meyer et al. 2012). Phage λ also requires at least four mutations to infect the novel OmpF receptor in *E. coli* B, making it almost impossible to evolve from the WT phage in a single step (Burmeister et al. 2016). Evolving all four mutations in one λ phage genome first required the presence of λ phage evolutionary intermediates that already carried specific mutations. These evolutionary intermediates may have arisen from the infection of easy-resistant *E. coli* B mutants (*malT* loss-of-function mutants) that could still spontaneously induce low levels of the traditional host receptor, LamB. By increasing the host diversity, phage λ eventually acquired additional mutations (four in total) to overcome the hard-resistant *E. coli B* mutants (*lamB* loss-of-function mutants) (Meyer et al. 2012; Gupta et al. 2022). Similarly, we showed that the successful infection of *waaG*, *galU*, *rfaH*, and *waaP*/*pssA* resistant strains became accessible by increasing host and phage diversity.

Our experiments suggest that it may be possible to breed well-established model phages such as ΦX174 to infect a wide range of currently resistant pathogenic *E. coli* strains. If possible, then this could make ΦX174 a promising therapeutic agent. Currently, instead of breeding well-known model systems to infect pathogenic bacteria, unknown phages that can infect specific bacterial pathogens are isolated from environmental samples (Chan et al. 2018). These unknown phages, however, still have to be characterized. Their safety has to be ensured by a process that, in the end, may drastically be more time-consuming than breeding well-studied model systems. Safety concerns regarding novel phages are significant due to their potential to carry dangerous toxins and antibiotic resistance genes (Krüger and Lucchesi 2015; Colavecchio et al. 2017; Jamet et al. 2017). The considerable knowledge acquired from decades of research on ΦX174 structure, life cycle, and evolution showed that ΦX174 does not carry virulence genes. ΦX174 is also highly host-specific (Michel et al. 2010), meaning that it will be harmless to the patient's microbiota, in contrast to phages with broader infectivity or antibiotics known to disrupt the microbiota and lead to adverse health outcomes (Ramirez et al. 2020). Furthermore, its use as a marker of immune responses in patients has already been approved in vivo by the Food and Drug Administration (FDA) in specific case studies (Rubinstein et al. 2000; Bearden et al. 2005). ΦX174 can easily be manipulated in a laboratory unlocking its potential as a powerful therapeutic agent. For all these reasons, we believe that bacteriophage ΦX174 could become a promising therapeutic agent.

## Materials and Methods

### Bacterial and Phage Strains

The ancestral *E. coli* strain C used in this study differs from the one uploaded on the National Center for Biotechnology Information (NCBI) website (GenBank accession number CP020543.1) by the presence of nine additional insertions and two nucleotide substitutions (c → t at the position 1,1720,214 and g → t at the position 1,190,560). The genomic sequence of CP020543.1 was manually modified using Geneious Prime (version 2020.1.2). All resistant bacterial strains generated in this study are derived from our ancestral *E. coli* C strain. All bacteriophage strains are derived from the ancestral coliphage ΦX174 (GenBank accession number AF176034). AF176034 genomic sequence was downloaded from the NCBI website and manually annotated using Geneious Prime (version 2020.1.2). Whole-genome resequencing on ΦX174 WT's glycerol stock showed no difference to the NCBI sequence. Complete genomes of *E. coli* C WT and ΦX174 WT used as references have been deposited in Zenodo (doi: 10.5281/zenodo.6952399). The *E. coli* C and ΦX174 ancestral strains were provided by Holly A. Wichman (Department of Biological Sciences, University of Idaho).

### Media and Growth Conditions

Phage and bacteria were grown in a shaking incubator (New Brunswick Innova 44) at 37°C, 250 rpm, in Lysogeny broth (LB, Miller) medium supplemented with CaCl_2_ and MgCl_2_ at a final concentration of 5 and 10 mm, respectively. Solid LB agar (1.5% agar) was used to plate both bacteria and phages. When plating phage, top agar overlay (0.5% agar), also called semisolid agar (SSA) in this study, was supplemented with CaCl_2_ and MgCl_2_ at a final concentration of 5 and 10 mm, respectively. Phage buffer juice solution (PBJ: 2.03% Tris-HCl, 0.61% MgCl_2_.6H_2_O) was used for serial dilutions. *E. coli* colony plates were generated by streaking with a sterile loop a scrap of the corresponding bacterial glycerol stock on the surface of a solid LB plate and incubated inverted overnight. After incubation, the colony plates were stored at 4°C for a maximum of two weeks. Overnight bacterial liquid cultures were systematically started by inoculating a single colony in 5 ml of LB supplemented with CaCl_2_ and MgCl_2_ at a final concentration of 5 and 10 mm, respectively, shaken at 250 rpm. Phage infections were either started from 1) a scrap of the corresponding phage glycerol stock resuspended in 100 μl of PBJ or 2) a single plaque isolated from the top agar overlay with a sterile cut tip (a circle of agar) directly inoculated inside the bacterial culture. Overnight incubations of both plates and cultures were set up for ∼16–17 h at 37°C.

### Measuring the Growth of Each Resistant *E. coli* C Strain

To assess the impact of LPS modifications harbored by the different *E. coli* C–resistant mutants on their growth, two independent cultures of each resistant strain were grown at 37°C, 250 rpm for 17 h. One hundred eighty μl of each overnight culture was inoculated into a 14-ml sterile tube (FALCON) containing 5 ml of LB (supplemented with CaCl_2_ and MgCl_2_ at final concentrations of 5 and 10 mm, respectively) prewarmed at 37°C. Incubation time was set to 6 h at 37°C, 250 rpm. Optical density at 600 nm (OD600) was measured every 30 min with an Ultrospec 10 (Biochrom). *E. coli* C WT was used as a reference. The mean OD600 values at each time point for each bacterial mutant were calculated and plotted.

### Isolation and Storage of *E. coli* C Strains with Resistance to WT ΦX174

To avoid competition and to produce a diverse set of phage-resistant bacterial mutants with different growth phenotypes, we generated ΦX174-resistant *E. coli* C strains on agar plates. Thirty-five *E. coli* C WT colonies were randomly chosen from an agar plate, and each was used to inoculate an independent overnight liquid culture. Fifty μl of each stationary-phase culture was mixed with 50 μl of a high titer (∼10^9^ plaque-forming unit [pfu] m/l) stock of ΦX174 WT lysate, immediately plated with sterile beads on LB plates, and incubated at 37°C overnight. From each plate, half a randomly chosen colony was used to inoculate a fresh overnight LB liquid culture. Once grown, a sample from each culture was mixed with glycerol saline solution and frozen at −80°C (giving strains *E. coli* C R1-R35). To confirm the resistance to ΦX174 WT, the remaining half of each colony was streaked onto an LB plate soaked with 100 μl of the high titer ΦX174 WT lysate and incubated at 37°C overnight.

### Phage Lysate Preparation

To extract phages in infected host cultures, 10 to 12 drops of chloroform were added to growing *E. coli* C–phage cocultures and vortexed for at least 30 s to kill the bacteria. Cultures were then centrifugated at 5,000 rpm for 10 min at 4°C. Supernatants (phage lysates) were transferred to sterile 5-ml Eppendorf tubes and stored at 4°C. One ml of each lysate was stored with glycerol saline solution and frozen at −80°C.

### First Phage Evolution Experiment: Standard

The protocol from Bono et al. (Bono et al. 201 3) was adapted to evolve phages that can infect resistant *E. coli* C strains. Of R1-R35—the 35 *E. coli* C strains resistant to ΦX174 WT, see above—31 were used for the first phage evolution experiment (excluding R1, R3, R15, and R19; see Results and supplementary text S1, Supplementary material online). Thirty-one ΦX174 lineages were each founded by ΦX174 WT and serially transferred daily, up to 21 days (21 transfers) in cultures containing a mix of *E. coli* C WT (permissive strain) and one resistant *E. coli* C strain (nonpermissive strain; R2-R35). For each transfer, permissive and nonpermissive bacteria were grown separately until they reached the log phase and then mixed in at a specific ratio depending on the growth of the nonpermissive strain (see fig. 3*A* and supplementary fig. S4 and methods, Supplementary Material online) to a final volume of 5 ml. At each transfer, freshly prepared susceptible cells were infected by ΦX174 at MOI_input_ ∼0.1. Infected cultures were shaken at 250 rpm, 37°C, for 3 h. Phage lysates were isolated as described in Phage lysate preparation. Phage lysates from the previous day were used to inoculate the fresh bacterial mixes the next day. Transfers continued until phages were found to infect their corresponding resistant *E. coli* C strain or until 21 transfers were reached. Three control lines were also run in parallel by transferring ΦX174 at MOI_input_ ∼0.1 in a culture containing *E. coli* C WT only. See supplementary methods, Supplementary Material online for a detailed version of the protocol.

### Second Phage Evolution Experiment: Increased Diversity

A phage cocktail made of 1) the WT ΦX174 and 2) all ΦX174 strains that successfully reinfected their corresponding resistant strains during the first phage evolution experiment was generated by first diluting each phage lysate and mixing them at a roughly equal number of pfu. Fourteen phage strains were used: ΦX174 R4 T7, ΦX174 R5 T2, ΦX174 R8 T6, ΦX174 R10 T6, ΦX174 R13 T6, ΦX174 R18 T4, ΦX174 R19 T1, ΦX174 R20 T5, ΦX174 R21 T1, ΦX174 R24 T1, ΦX174 R26 T1, ΦX174 R27 T1, ΦX174 R29 T1, and ΦX174 R31 T1. Phages infecting R5 and R19 were used for the cocktail but removed from the mutational analysis (see supplementary text S1 and table S4, Supplementary Material online). The phage cocktail was grown and transferred daily for up to four days, on nonevolving host cultures containing 1) *E. coli* C WT, 2) the 14 host strains (see supplementary text S1 and table S4, Supplementary Material online) for which resistance had been overcome (permissive hosts), and 3) an excess of one of the still-resistant strain of interest (nonpermissive host). For the nonpermissive host, we used R6 (*waaP/pssA* mutant), R22 (*galU* mutant), R25 (*rfaH* mutant), or R28 (*waaG* mutant; see supplementary table S1, Supplementary Material online). *E. coli* C R22 and R28 were chosen as representatives of the *galU* and *waaG* mutants, respectively. Each bacterial strain (permissive and nonpermissive) was grown separately in LB liquid culture for 1 h (37°C, 250 rpm) and then pooled at a roughly equal number of colony-forming units. The resistant strain of interest was added in excess to give a final volume of 4 ml. Infected cultures were initiated by adding the phage cocktail (MOI_input_ ∼0.1), shaken at 250 rpm, 37°C, for 5 h (see fig. 3*B*). Phage lysates were isolated as described in Phage lysate preparation. Phage lysates from the previous day were used to inoculate the fresh bacterial mixes the next day. This process was repeated until phages were found to infect their corresponding resistant *E. coli* C strains or until four transfers had been completed. See supplementary methods, Supplementary Material online for a detailed version of the protocol.

### Third Evolution Experiment: Increased Diversity and Generations

Protocol from the second evolution experiment was adjusted to reduce the amount of time necessary to retrieve the desired phages. The same phage cocktail used in the second phage evolution was grown and transferred daily, four times a day, for up to four days on nonevolving host cultures containing 1) *E. coli* C WT, 2) the 14 host strains for which resistance had been overcome (permissive hosts), and 3) one of the still-resistant strain of interest (nonpermissive host). We increased the number of serial transfers per day from one to four, for a total of four days (16 transfers). *E. coli* C R6 (*waaP/pssA* mutant) and R25 (*rfaH* mutant, see supplementary table S1, Supplementary Material online) were used as nonpermissive hosts. After reaching their exponential log phase, bacterial strains (except the nonpermissive hosts) were mixed depending on their growth (fig. 3*C* and supplementary fig. S4 and methods, Supplementary Material online). New host cultures were kept in exponential growth phase independently by transferring every hour 1:5 of the volume (1 ml) in fresh LB preheated at 37°C (see fig. 3*C*). Before each transfer, all host cultures were mixed together. Infected cultures were started by adding the phage cocktail to the final host mix at MOI_input_ ∼0.1, shaken at 250 rpm, 37°C, for 1 h. After that, 1:15 of the volume of each infected culture was transferred in freshly mixed bacterial cultures and infection continued for 1 h (see fig. 3*C*). This step was repeated for a total of four transfers. The final (fourth) transfer lasted for 2 h. All transfers completed on the same day involved transferring both phage and bacteria; on every fourth transfer, supernatants were collected, and only phages were transferred. Phage lysates were isolated as described in Phage lysate preparation. Phage lysates from the previous day were used to inoculate the first fresh bacterial mixes the next day. This process was repeated until phages were found to infect their corresponding resistant *E. coli* C strains or until four transfers had been completed. See supplementary methods, Supplementary Material online for a detailed version of the protocol.

### Isolation of Evolved ΦX174 Strains from Evolution Experiments

To isolate pure, single clones from the different evolution experiments, top agar overlays were prepared by mixing 100 μl of undiluted phage lysates with 200 μl of an overnight culture of the corresponding resistant *E. coli* C strain in 4 ml SSA. Plates were incubated inverted at 37°C for ∼16–17 h. An isolated plaque was chosen randomly for each phage lysate to infect cultures of the corresponding resistant *E. coli* C strains (in exponential growth state) incubated at 37°C, 250 rpm, for 5 h. Phage lysates were isolated as described in Phage lysate preparation. Phage isolates were reisolated from single plaques a second time from their glycerol stocks. See supplementary methods, Supplementary Material online for a detailed version of the protocol.

### Determination of the Evolved Phages’ Host Range by Spotting Assays

#### Method 1

Each top agar overlay was prepared by mixing 200 μl of an *E. coli* C strain from stationary-phase culture in 4 ml SSA, then poured on LB plates, and dried for at least 15 min. Then, 3 μl of each undiluted evolved phage lysate (between 10^7^ and 10^9^ pfu/ml) was dropped at the surface.

#### Method 2

Each top agar overlay was prepared by mixing a volume of each phage lysate in 4 ml SSA at a final concentration of ∼10^7^ pfu/ml, then poured on LB plates, and dried for at least 15 min. Then, 3 μl of both undiluted and 10-fold diluted of each *E. coli* C strain (from overnight culture) were dropped onto the surface with a pipette. For both methods, spots were dried for at least 30 min, and plates were incubated inverted at 37°C for ∼17 h. *E. coli* C WT was used as a positive control (permissive strain) and *E. coli* K-12 MG1655 as a negative control (nonpermissive strain). A bacterial host strain was classified as sensitive only when signs of lysis were detected using both methods in at least two (of three) replicates per method. A phage–bacterium combination that yielded different results between the two methods was tested in standard plaque assays. Finally, the host strain was classified as sensitive if plaques were observed at both dilutions. See supplementary methods, Supplementary Material online for a detailed version of the protocol.

### Data Visualization

The hierarchical agglomerative clustering analysis (clustermap; fig. 6), the heatmap (supplementary fig. S3, Supplementary Material online), and the growth curves (supplementary fig. S4, Supplementary Material online) were generated using the default settings of the Seaborn library (version 0.11.2) for Python (version 3.7.4). All schematic drawings (figs. 1–4 and supplementary figs. S1 and S2, Supplementary Material online) were created with BioRender.com. The Lollipop plot (fig. 5*A*) was generated using trackViewer Vignette: lollipopPlot (Lolliplot) in R (version 1.27.11).

### Whole-Genome Resequencing

#### Bacteria

Samples were prepared for whole-genome resequencing from 1 ml of stationary-phase culture. Genomic DNA was extracted using the Wizard Genomic DNA Purification Kit (Promega, Germany). Extracted DNA was tested for quality, pooled, and sequenced by the Max Planck Institute for Evolutionary Biology (Plön, Germany) using an Illumina Nextera DNA Flex Library Prep Kit to produce 150 bp paired-end reads (Picelli et al. 2014).

#### Phage

ΦX174 samples were prepared for whole-genome resequencing from 1 ml of phage lysate. Genomic DNA was extracted using the QIAprep Spin Miniprep Kit (Qiagen) and then amplified by performing 20 cycles of PCR using Q5 High-Fidelity 2X Master Mix (NEB) (final concentration between 30 and 100 ng/µl). The primers used for the amplification of the ΦX174 genome are listed in supplementary table S6, Supplementary Material online. All PCR products were cleaned using the QIAquick PCR Purification Kit (Qiagen). DNA samples were tested for quality, pooled, and sequenced by the Max Planck Institute for Evolutionary Biology (Plön, Germany). Sequencing was performed using an Illumina MiSeq DNA Flex Library Prep Kit to produce 150 bp paired-end reads (Picelli et al. 2014).

The quality of the sequencing output from bacteria and phages was controlled using *FastQC* version 0.11.8. Reads were trimmed using *Trimmomatic*, assembled, and analyzed using the *breseq* pipeline version 0.33.2 (Barrick et al. 2014; Deatherage and Barrick 2014; Deatherage et al. 2015) and Geneious Prime (version 2020.1.2).

### Sanger Sequencing of ΦX174 *F* and *H* Genes

Genomic DNA was extracted using the QIAprep Spin Miniprep Kit (Qiagen) (final concentration between 30 and 100 ng/µl). ΦX174's *F* and *H* genes were amplified by performing 35 cycles of PCR using Phusion High-Fidelity PCR Master Mix with HF Buffer (ThermoFisher). Primers used for this purpose are listed in supplementary table S6, Supplementary Material online. All PCR products were cleaned using the QIAquick PCR Purification Kit. Sanger sequencing was performed using the sequencing primers listed in supplementary table S6, Supplementary Material online. The sequencing was performed at the Max Planck Institute for Evolutionary Biology (Plön, Germany). Sequencing results were assembled and analyzed with Geneious Prime (version 2020.1.2).

## Supplementary Material

msad154_Supplementary_Data

## Data Availability

Complete genomes of *E. coli* C WT and ΦX174 WT used as references and raw sequencing reads used to generate supplementary tables S1 to S5, Supplementary Material online have been deposited in Zenodo (doi: 10.5281/zenodo.6952399). *Growth curves.* Raw OD measurements used to generate supplementary figure S4, Supplementary Material online have been deposited in Zenodo (doi: 10.5281/zenodo.6952399). *Hierarchical agglomerative clustering and heatmap data.* Pictures and raw data used to generate fig 6 and Supplementary fig. S3, Material online have been deposited in Zenodo (doi: 10.5281/zenodo.6952399).
