# Supplementary material for: Stepwise Evolution of E. coli C and ΦX174 Reveals Unexpected Lipopolysaccharide (LPS) Diversity
Source: Mol Biol Evol. 2023 Jul 3;40(7):msad154. doi: 10.1093/molbev/msad154 (PMC10368449; doi:10.1093/molbev/msad154)
Supplement: msad154_Supplementary_Data [file msad154_supplementary_data.pdf]

Supplementary Text S1

Excluded bacteria and phage isolates from downstream analyses. We removed four bacterial strains (*E. coli* C R1, R3, R15, and R19) and two evolved phage strains (ΦX174 R5 T2 and ΦX174 R19 T1) from both mutational and phenotypical analyses. The reasons behind these exclusions are outlined below.

Bacteria. We found that both *E. coli* C R3 and R15's glycerol stocks contain more than one genotype; while whole genome re-sequencing from their respective glycerol stocks showed only a single mutation in *galE*, whole genome re-sequencing of ten re-streaked colonies showed that additional mutations were systematically associated with the single mutation in *galE* (**Tables S4 and S5**). *E. coli* C R1 displayed an unstable resistant phenotype using the spotting assay *method 1* (see section **Determination of the evolved phages' host range by spotting assays**). We performed phenotypic assays in a semi-solid environment by plating top agar overlays from overnight cultures of *E. coli* C R1 without phage. All overnight cultures were started with a randomly picked single colony obtained from the glycerol stock of R1. We observed that colonies of R1 made different lawn types: either "smooth" (no bacterial aggregate) or "granulous" (presence of numerous bacterial aggregates), which could potentially impact phage infectivity. The existence of two different aggregation phenotypes suggests that the glycerol stock of *E. coli* C R1 consists of two different populations. Unlike R3 and R15, however, no discrepancy was found between the whole genome re-sequencing results of R1 from the glycerol stock and its ten re-streaked colonies. The exact cause of the phenotypic inconsistency could not be identified. *E. coli* C R19 remains sensitive to ΦX174 wildtype infection. When plated undiluted, a high titer ΦX174 wildtype lysate (~10^9 pfu ml^{-1}) could not clear *E. coli* C R19's lawn, but a few thousand small clear plaques were produced. Thus, R19 is likely to be only partially resistant to ΦX174 wildtype infection. It carries a single mutation *yajC* (locus tag B6N50_17610), which encodes a periplasmic protein (Fang and Wei 2011) with a putative preprotein translocase subunit (Pfam e-value 2.3e-26) (see **Table S4**). While no link to the LPS biosynthesis or assembly has been defined yet, *yajC* could conceivably play a role in the injection of phage DNA into the bacterium's cytoplasmic membrane (Schulze et al. 2014; Bohm et al. 2018).

In our matrices (**Figs. 6 and S3**), *E. coli* C R32 cannot be infected by any evolved phages from the first evolution experiment. The phage infecting R5 was the only phage that could infect R32 but was removed from the subsequent analysis (see below).

Phage. We removed the evolved phage infecting R5 (ΦX174 R5 T2) because its glycerol stock contains more than one genotype (confirmed by Sanger Sequencing). Since we removed R19 from the final analysis, we also removed its corresponding evolved phage obtained during the first evolution experiment (ΦX174 R19 T1). Details on their respective mutations (in the *H* gene) can be found in **Table S4**.

Supplementary Figures

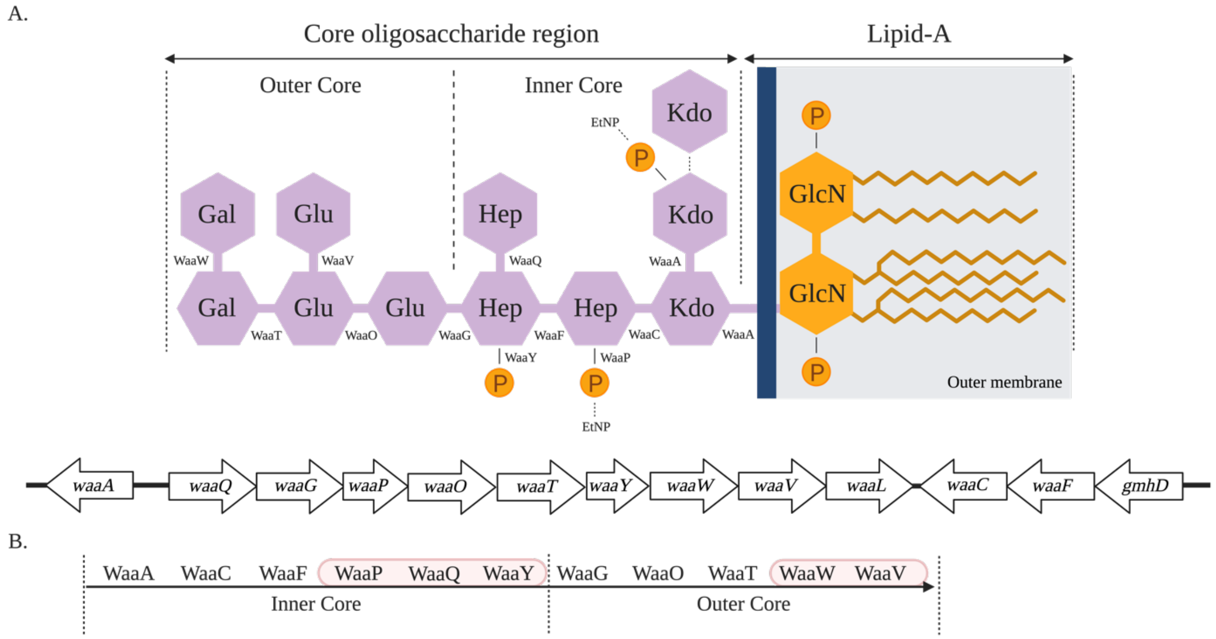

Fig. S1. Assembly of *E. coli* rough type core LPS. A. Organization of the chromosomal *waa* region in the *E. coli C* wildtype strain. The chromosomal *waa* locus (formerly named *rfa*) is organized into three major operons, usually designated by the first gene of each transcriptional unit: *waaA, gmhD* and *waaQ* (Whitfield et al. 1999). The *waaA* operon is responsible for the incorporation of two Kdo (3-deoxy-D-*manno*-octulosonic acids) moieties to the lipid A (Belunis et al. 1995). The *gmhD* operon is required for the assembly of the inner core's backbone (Schnaitman and Klena 1993), and the *waaQ* operon contains all eight genes necessary to modify the inner core and built the outer core (Whitfield et al. 1999) **B.** Sequential steps of the core OS LPS synthesis (from WaaA to WaaV). *Inner core assembly and modification*. Inner core assembly starts first with WaaA adding the two Kdo moieties to the lipid-A-IV precursor (Schnaitman and Klena 1993; Whitfield et al. 1999; Amor et al. 2000). A bacterium lacking WaaA activity displays severe membrane defects, resulting in the inability to form colonies (Klein et al. 2009). Heptose residues I and II are then anchored to the Kdo moieties by the heptosyltransferases WaaC and WaaF, respectively (Jansson et al. 1981; Schnaitman and Klena 1993; Whitfield et al. 1999). LPS core heptose kinases WaaP and WaaY add phosphate groups to the first and second heptose residues, respectively. WaaQ adds the last heptose to the second heptose residue (Jansson et al. 1981; Schnaitman and Klena 1993; Whitfield et al. 1999). *WaaP*, *waaQ*, and *waaY* proceed in this specific order. *WaaY* cannot work without the activity of *waaQ*, which cannot work

without *waaP* (first red ellipse). *Outer core assembly.* The glucosyltransferases WaaG and WaaO start the formation of the outer core by anchoring the first glucose to the second heptose residue and the second glucose to the first glucose residue, respectively (Heinrichs, Yethon, and Whitfield 1998; Vinogradov et al. 1999; Whitfield et al. 1999). It has been suggested that the WaaG-catalyzed reaction might be required for WaaP and WaaY substrate specificity (Yethon et al. 2000). Then, the galactosyltransferases WaaT and WaaW add the first galactose to the second glucose residue and the second galactose to the first galactose residue, respectively (Heinrichs, Yethon, Amor, et al. 1998; Whitfield et al. 1999). The completion of the outer core LPS is achieved when WaaV adds the third glucose to the second glucose residue. Here, *waaV* requires the activity of *waaW* and proceeds in this specific order (second red ellipse). If *waaW* is deleted, *waaV* is not functional (Heinrichs, Yethon, Amor, et al. 1998; Leipold et al. 2007). Modifications of the LPS structure can lead to major phenotypic effects. In particular, the deep rough phenotype is usually associated with a strongly destabilized outer membrane, a decreased expression of some outer membrane proteins, a modification of the turgor pressure (Pagnout et al. 2019), and an increase of susceptibility to hydrophobic compounds such as AMPs (antimicrobial peptides), antibiotics, or bacteriocins (van der Ley et al. 1986; Schnaitman and Klena 1993; Yethon et al. 1998; Whitfield et al. 1999; Amor et al. 2000; Klein et al. 2013). Differences in LPS structures can also affect interactions with the host immune system (Raetz and Whitfield 2002; Matsuura 2013) and phage resistance (Hancock and Reeves 1976; Labrie et al. 2010; Kulikov et al. 2019; Mutalik et al. 2020).

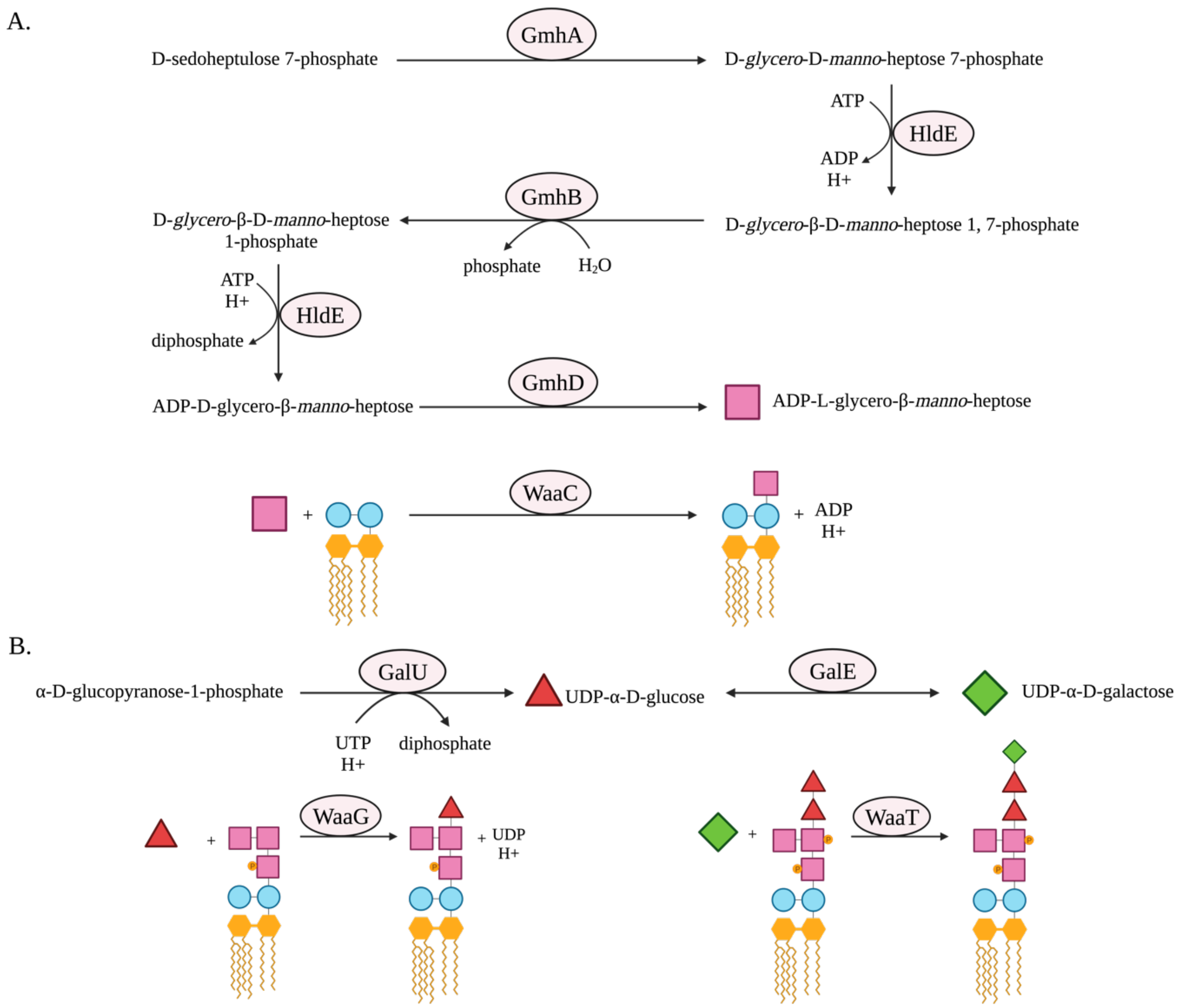

Fig S2. Biosynthesis of the core LPS glucose, galactose and heptose sugar components. A. ADP-L-glycero-β-D-*manno*-heptose synthesis pathway. It involves four genes: *gmhA*, *hldE*, *gmhB* and *gmhD*. Each gene encodes a protein that catalyses the production of a heptose intermediate. The final product is ADP-L-glycero-β-D-*manno*-heptose, which is used by the heptosyltransferase WaaC to build the inner core LPS (Kneidinger et al. 2002). Thus, deletion of one of these genes is expected to result in an heptoseless, deep rough LPS (McArthur et al. 2005). This figure is adapted from https://biocyc.org/ECOLI/NEW-IMAGE?type=PATHWAY&object=PWY0-1241 (Karp et al. 2019). **B.** Galactose degradation I (Leloir Pathway). GalU and GalE function in the Leloir pathway (Frey 1996; Kneidinger et al. 2002; McArthur et al. 2005). GalU catalyses the formation of UDP-α-D-glucose from α-D-glucopyranose 1- phosphate (Weissborn et al. 1994). UDP-α-D-glucose can then be incorporated into the outer core LPS by WaaG. Therefore, both Δ*galU* and Δ*waaG* mutants result

in the same truncated LPS structure (Schnaitman and Klena 1993; Weissborn et al. 1994; Genevaux et al. 1999). GalE catalyses the interconversion of UDP-α-D-galactose and UDP-α-D-glucose during galactose catabolism (Pierson and Carlson 1996). UDP-α-D-galactose is then incorporated into the outer core LPS by WaaT (Heinrichs, Yethon, Amor, et al. 1998). Therefore, both Δ*galE* and Δ*waaT* are expected to result in the same truncated LPS (Schnaitman and Austin 1990). This figure was adapted from https://biocyc.org/ECOLI/NEW-IMAGE?type=PATHWAY&object=GALACTMETAB-PWY (Karp et al. 2019).

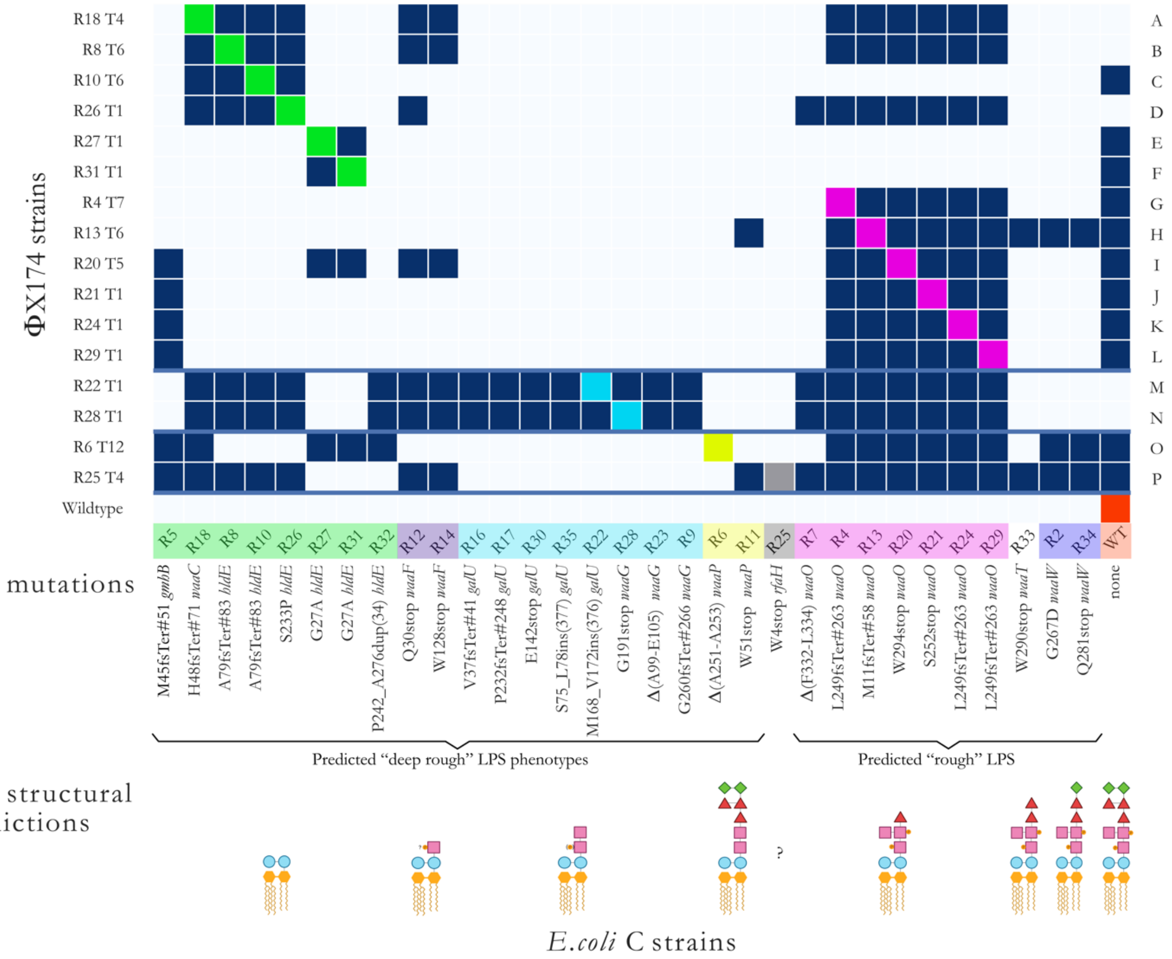

Fig. S3. Infection matrix of evolved ΦX174 phages on the 31 resistant *E. coli* C strains. The infection matrix was produced by combining the results of two spotting methods and the plaque assays (see **Methods**). *E. coli* C R32 cannot be infected by any evolved phages from the first phage evolution experiment. The phage infecting R5 was the only phage that could infect R32 (**Text S1**) but was removed from the subsequent analysis due to a lack of isogeny. *E. coli* C strains are grouped and coloured based on their predicted core LPS structures (Jansson et al. 1981; Schnaitman and Austin 1990; Schnaitman and Klena 1993; Weissborn et al. 1994; Heinrichs, Yethon, and Whitfield 1998; Genevaux et al. 1999; Vinogradov et al. 1999; Whitfield et al. 1999; Amor et al. 2000; Kawaura et al. 2000; Yethon et al. 2000; Kneidinger et al. 2002; Raetz and Whitfield 2002; McArthur et al. 2005; Leipold et al. 2007; Fang and Wei 2011; Król et al. 2019). ΦX174 strains are ordered based on the predicted LPS structures they infected during the evolution experiments (A-F: overcame heptoseless *waaC* and *hldE* mutants; G-L: overcame *waaO* mutants; M-N: overcame *galU* and *waaG* mutants; O:

overcame *waaP/pssA* mutant; P: overcame *rfaH* mutant). The solid blue lines separate the evolved phages according to the evolution experiment in which they were isolated (A-L: first; M-N: second; O-P: third). Dark blue squares=infection, light blue square=no infection, coloured square=control infection by a phage evolved on that host. R# indicates the number of the resistant strain the phage evolved on, and T# is the transfer number where plaques were first observed. "?": core LPS structure of *E. coli* C R25 (*rfaH* mutant) could not be predicted. "INS": insertion. "DUP": duplication. "stop": stop codon. "Δ": deletion. Examples: L249fsTer#263 indicates a frameshift (fs) leading to a premature codon stop (Ter); the position of the premature stop codon is in parentheses. S75_L78ins(377) indicates an insertion; the two flanking amino acids are separated by a "_" and followed by the number of inserted amino acids in parentheses. Δ(A99-E105) indicated deletion; two flanking amino acids are separated by a "-". P242_A276dup(34) indicates a duplication; the two flanking amino acids are separated by a "_" and followed by the number of duplicated amino acids in parentheses.

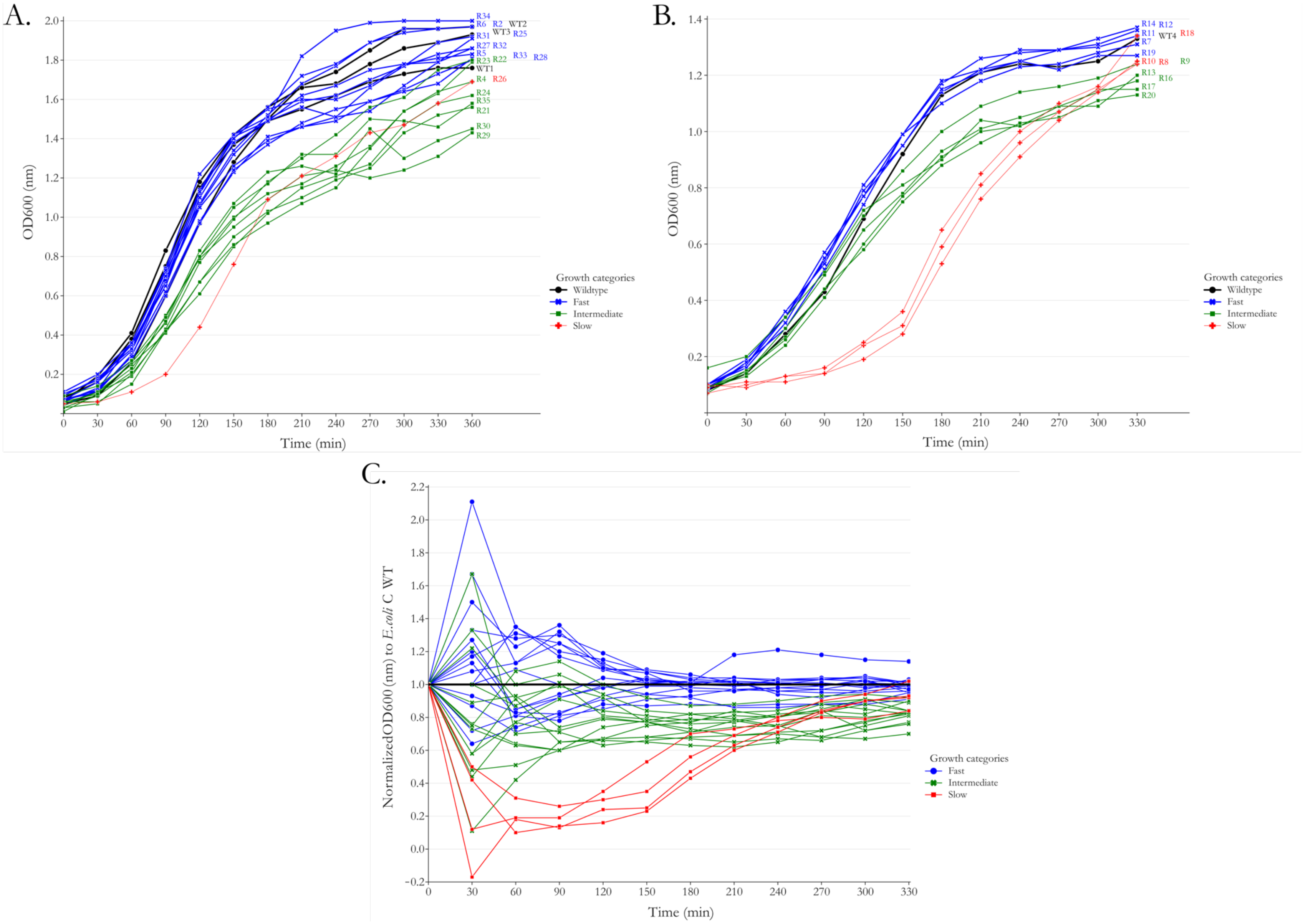
A.
OD600 (nm)
Time (min)
R34
R6
R2
WT2
WT3
R25
R31
R27
R32
R5
R33
R28
R23
R22
WT1
R4
R26
R24
R35
R21
R30
R29
Growth categories
Wildtype
Fast
Intermediate
Slow
B.
OD600 (nm)
Time (min)
R14
R12
R11
WT4
R18
R7
R19
R10
R8
R9
R13
R16
R17
R20
Growth categories
Wildtype
Fast
Intermediate
Slow
C.
NormalizedOD600 (nm) to E.coli C WT
Time (min)
Growth categories
Fast
Intermediate
Slow

Fig. S4. *E. coli* C resistant strains can be categorized as fast, intermediate, or slow growers. The mean OD600 values of two independent cultures grown in 5 ml LB at each time point for each bacterial mutant were calculated and plotted. Resistant strains are categorized with respect to their growth compared to *E. coli* C wildtype. Black lines: *E. coli* C wildtype. Blue lines: resistant bacteria that grew similarly to *E. coli* C wildtype ("fast growers"). Green lines: resistant strains grew somewhat more slowly than *E. coli* C wildtype and fast growers ("intermediate growers"). Red lines: resistant strains that grew more slowly than *E. coli* C wildtype and intermediate growers ("slow growers"). **A.** Growth curves of resistant strains R2-R6 and R21-R35. **B.** Growth curves of resistant strains R7-R20. These data (mutants and controls) were collected in a separate block to those in panel A. **C.** Mean OD600 values of all resistant strains in panels A and B, each normalized with respect to their corresponding *E. coli* C wildtype control ((OD600_R#$_{ti}$ – ODR#$_{t0}$)/(OD600_wildtype$_{ti}$ – OD600_wildtype$_{t0}$)). R# is the resistant strain number.

E. coli C resistant strains	Genes	Locus tags	Synonyms	Descriptions	Positions	Nucleotide changes	Amino Acid changes	Predicted LPS structure phenotypes	"Easy" or "Hard" LPS structure phenotypes?	# Experiment during which resistance was overcome or cross-infected
R2	*waaW*	*B6N50_00430* →	-	UDP-galactose--(galactosyl) LPS alpha1,2-galactosyltransferase	79,779	g→a	G267D	Rough	Easy	1
R4	*waaO*	*B6N50_00415* →	*rfaI*	UDP-D-glucose:(glucosyl) LPS α-1,3-glucosyltransferase	76,970	$(a)_{7\rightarrow6}$	L249fsTer#263[a]	Rough	Easy	1
R5	*gmhB*	*B6N50_18960* ←	-	D-glycero-β-D-manno-heptose 1,7-bisphosphate 7-phosphatase	3,693,747	$(t)_{8\rightarrow7}$	M45fsTer#51	Deep rough	Easy	1
R6	*waaP*	*B6N50_00410* →	*rfaP*	Lipopolysaccharide core heptose(I) kinase	76,163 - 76,168	Δ6 bp	Δ(A251-A253)	Deep rough	Hard	3
	pssA	*B6N50_06070* ←	-	Phosphatidylserine synthase	1,190,560	t→g	V54V			
R7	*waaO*	*B6N50_00415* →	*rfaI*	UDP-D-glucose:(glucosyl) LPS α-1,3-glucosyltransferase	77,219 - 77,224	$(ttattt)_{2\rightarrow1}$	Δ(F332-L334)	Rough	Easy	1
R8	*hldE*	*B6N50_03700* →	*rfaE/waaE*	Bifunctional heptose 7-phosphate kinase/heptose 1-phosphate adenyltransferase	721,891 - 721,892	$(gc)_{5\rightarrow4}$	A79fsTer#83	Deep rough	Easy	1
R9	*waaG*	*B6N50_00405* →	*rfaG*	UDP-glucose:(heptosyl) LPS α1,3-glucosyltransferase (glucosyltransferase I)	75,073 - 75,076	Δ4 bp	G260fsTer#266	Deep rough	Hard	2
R10	*hldE*	*B6N50_03700* →	*rfaE/waaE*	Bifunctional heptose 7-phosphate kinase/heptose 1-phosphate adenyltransferase	721,891 - 721,892	$(gc)_{5\rightarrow4}$	A79fsTer#83	Deep rough	Easy	1
	CRISPR	*B6N50_05180* → / ← *B6N50_05185*	-	Repeat region	1,023,697 - 1,023,940	Δ244 bp	Non-coding region			
R11	*waaP*	*B6N50_00410* →	*rfaP*	Lipopolysaccharide core heptose(I) kinase	75,562	g→a	W51stop	Deep rough	Easy	1

R12	*waaF*	*B6N50_00450* ←	*rfaF*	ADP-heptose LPS heptosyltransferase II	84,376	g→a	Q30stop	Deep rough	Easy	1
R13	*waaO*	*B6N50_00415* →	*rfaI*	UDP-D-glucose:(glucosyl) LPS α-1,3-glucosyltransferase	76,255	Δ1 bp	M11fsTer#58	Rough	Easy	1
R14	*waaF*	*B6N50_00450* ←	*rfaF*	ADP-heptose LPS heptosyltransferase II	84,081	c→t	W128stop	Deep rough	Easy	1
R16	*galU*	*B6N50_13325* ←	-	UTP--glucose-1-phosphate uridylyltransferase	2,611,994	Δ1 bp	V37fsTer#41	Deep rough	Hard	2
R17	*galU*	*B6N50_13325* ←	-	UTP--glucose-1-phosphate uridylyltransferase	2,611,409	$(g)_{4\rightarrow3}$	P232fsTer#248	Deep rough	Hard	2
R18	*waaC*	*B6N50_00445* ←	*rfaC*	ADP-heptose LPS heptosyltransferase I	83,271	Δ1 bp	H48fsTer#71	Deep rough	Easy	1
R20	*waaO*	*B6N50_00415* →	*rfaI*	UDP-D-glucose:(glucosyl) LPS α-1,3-glucosyltransferase	77,104	g→a	W294stop	Rough	Easy	1
R21	*waaO*	*B6N50_00415* →	*rfaI*	UDP-D-glucose:(glucosyl) LPS α-1,3-glucosyltransferase	76,978	c→a	S252stop	Rough	Easy	1
	potH	*B6N50_15295* ←	-	Spermidine/putrescine ABC transporter permease	2,990,409	g→t	Q310K			
R22	*galU*	*B6N50_13325* ←	-	UTP--glucose-1-phosphate uridylyltransferase	2,611,590 - 2,611,600	INS 2,783,815 - 2,784,943	M168_V172ins(376)[b]	Deep rough	Hard	2
R23	*waaG*	*B6N50_00405* →	*rfaG*	UDP-glucose:(heptosyl) LPS α1,3-glucosyltransferase (glucosyltransferase I)	74,589 - 74,606	Δ18 bp	Δ(A99-E105)	Deep rough	Hard	2
R24	*waaO*	*B6N50_00415* →	*rfaI*	UDP-D-glucose:(glucosyl) LPS α-1,3-glucosyltransferase	76,970	$(a)_{7\rightarrow6}$	L249fsTer#263	Rough	Easy	1
R25	*rfaH*	*B6N50_22865* →	-	Transcription/translation regulatory transformer protein	4,485,876	g→a	W4stop	Unknown	Hard	3
R26	*hldE*	*B6N50_03700* →	*rfaE/waaE*	Bifunctional heptose 7-phosphate kinase/heptose 1-phosphate adenyltransferase	722,353	t→c	S233P	Deep rough	Easy	1

R27	*hldE*	*B6N50_03700* →	*rfaE/waaE*	Bifunctional heptose 7-phosphate kinase/heptose 1-phosphate adenyltransferase	721,736	g→c	G27A	Deep rough	Easy	1
R28	*waaG*	*B6N50_00405* →	*rfaG*	UDP-glucose:(heptosyl) LPS α1,3-glucosyltransferase (glucosyltransferase I)	74,864	g→t	G191stop	Deep rough	Hard	2
R29	*waaO*	*B6N50_00415* →	*rfaI*	UDP-D-glucose:(glucosyl) LPS α-1,3-glucosyltransferase	76,970	$(a)_{7\rightarrow6}$	L249fsTer#263	Rough	Easy	1
	pcoA	*B6N50_01165* ←	-	Multicopper oxidase	237,963 - 237,973	INS 1,040,936 - 1,042,273	G152_G155ins(446)			
	eutM - *eutN* - *eutE*	*[B6N50_06785]* - *[B6N50_06790]* - *[B6N50_06795]* →	-	Ethanolamine utilization protein EutM - Ethanolamine utilization protein EutN - Aldehyde dehydrogenase EutE	1,342,381 - 1,343,448	Δ1,068 bp	Δ[EutM_A58-EutE_A181][c]			
R30	*galU*	*B6N50_13325* ←	-	UTP--glucose-1-phosphate uridylyltransferase	2,611,680	c→a	E142stop	Deep rough	Hard	2
R31	*hldE*	*B6N50_03700* →	*rfaE/waaE*	Bifunctional heptose 7-phosphate kinase/heptose 1-phosphate adenyltransferase	721,736	g→c	G27A	Deep rough	Easy	1
R32	*hldE*	*B6N50_03700* →	*rfaE/waaE*	Bifunctional heptose 7-phosphate kinase/heptose 1-phosphate adenyltransferase	722,381 - 722,482	DUP 722,381-722,482 (102) bp	P242_A276dup(34)[d]	Deep rough	Easy	1
R33	*waaT*	*B6N50_00420* →	-	UDP-galactose:(glucosyl) LPS α-1,2-galactosyltransferase	78,125	g→a	W290stop	Rough	Easy	1
R34	*waaW*	*B6N50_00430* →	-	UDP-galactose--(galactosyl) LPS alpha1,2-galactosyltransferase	79,820	c→t	Q281stop	Rough	Easy	1
R35	*galU*	*B6N50_13325* ←	-	UTP--glucose-1-phosphate uridylyltransferase	2,611,872 - 2,611,880	INS 2,783,815 - 2,784,945	S75_L78ins(377)	Deep rough	Hard	2

Table S1: List of mutations found in *E. coli* C strains that are resistant to wildtype ΦX174. *E. coli* C resistant strains: R# is the strain number. Genes: name of the gene(s) in which mutations have been identified (as compared with wildtype *E. coli* C; doi: 10.5281/zenodo.6952399). Locus tags: identifier of each listed gene. Synonyms: alternative gene names. Descriptions: protein product encoded by each listed gene. Positions: genomic coordinates of mutation. Nucleotide changes: observed nucleotide change. Amino acid changes: resulting change in amino acid sequence. Predicted LPS structure phenotypes: based on predicted LPS structure (see **Fig. 2**). “Easy” or “Hard” LPS structure phenotypes?: easy LPS phenotypes were overcome or could be cross-infected after the first phage evolution experiment, while hard LPS phenotypes required the second or third phage evolution experiment to be infected. “INS”: insertion. “DUP”: duplication. “stop”: stop codon. “[a]”: L249fsTer#263 indicates a frameshift (fs) leading to a premature codon stop (Ter); the position of the premature stop codon is in parentheses. “[b]”: M168_V172ins(376) indicates an insertion; the two flanking amino acids are separated by a “_” and followed by the number of inserted amino acids in parentheses. “[c]”: Δ[EutM_A58-EutE_A181] indicated a long deletion starting from EutM to EutE. P242_A276dup(34) indicates a duplication; the two flanking amino acids are separated by a “_” and followed by the number of duplicated amino acids in parentheses.

ΦX174 strains	Evolution experiment #	Gene mutated in the corresponding *E. coli* C resistant strains	Mutants sensitive to the evolved ΦX174 strain infection	Gene mutated in ΦX174 strains	Descriptions	Positions	Nucleotide changes	Amino acid changes	Are mutations present in a control lineage?
R21 T1	1	*waaO*	R5, R4, R13, R20, R21, R24, R29, WT	*F*	Capsid protein	1,347	a→g	D116G	No
				H	Minor spike protein	3,132	g→a	A68T	No
R24 T1	1	*waaO*	R5, R4, R13, R20, R21, R24, R29, WT	*F*	Capsid protein	1,346	g→a	D116S	No
				F	Capsid protein	1,347	a→g	D116S	No
R26 T1	1	*hldE*	R18, R8, R10, R26, R12, R7, R4, R13, R20, R21, R24, R29	*F*	Capsid protein	1,460	c→a	Q154K	No
				H	Minor spike protein	3,132	g→a	A68T	No
R27 T1	1	*hldE*	R27, R31, WT	*H*	Minor spike protein	3,202	g→t	G91V	No
R29 T1	1	*waaO*	R5, R4, R13, R20, R21, R24, R29, WT	*F*	Capsid protein	1,346	g→a	D116S	No
				F	Capsid protein	1,347	a→g	D116S	No
R31 T1	1	*hldE*	R27, R31, WT	*H*	Minor spike protein	3,202	g→t	G91V	No
R18 T4	1	*waaC*	R18, R8, R10, R26, R12, R14, R4, R13, R20, R21, R24, R29	*F*	Capsid protein	1,307	t→c	Y103H	No
				F	Capsid protein	1,461	a→g	Q154R	No
				H	Minor spike protein	3,132	g→a	A68T	No
R20 T5	1	*waaO*	R5, R27, R31, R12, R14, R4, R13, R20, R21, R24, R29, WT	*F*	Capsid protein	1,354	t→a	N118K	No
				H	Minor spike protein	3,132	g→a	A68T	No
R8 T6	1	*hldE*	R18, R8, R10, R26, R12, R14, R4, R13, R20, R21, R24, R29	*F*	Capsid protein	1,307	t→c	Y103H	No
				F	Capsid protein	1,461	a→g	Q154R	No
				F	Capsid protein	3,132	g→a	A68T	No
R10 T6	1	*hldE*	R18, R8, R10, R26, WT	*F*	Capsid protein	1,317	a→g	H106R	No

				F	Capsid protein	1,460	c→a	Q154K	No
R13 T6	1	*waaO*	R11, R4, R13, R20, R21, R24, R29, R33, R2, R34, WT	*F*	Capsid protein	2,015	g→c	D339H	No
R4 T7	1	*waaO*	R4, R13, R20, R21, R24, R29, WT	*F*	Capsid protein	1,301	a→g	T101A	Yes (in C1 T21)
				F	Capsid protein	1,347	a→g	D116G	No
				F	Capsid protein	2,085	c→t	A362V	No
R22 T1	2	*galU*	R18, R8, R10, R26, R32, R12, R14, R16, R17, R30, R35, R22, R28, R23, R9, R7, R4, R13, R20, R21, R24, R29	*F*	Capsid protein	1,307	t→c	Y103H	No
				F	Capsid protein	1,317	a→g	H106R	No
				F	Capsid protein	1,461	a→g	Q154R	No
				H	Minor spike protein	3,132	g→a	A68T	No
R28 T1	2	*waaG*	R18, R8, R10, R26, R32, R12, R14, R16, R17, R30, R35, R22, R28, R23, R9, R7, R4, R13, R20, R21, R24, R29	*F*	Capsid protein	1,307	t→c	Y103H	No
				F	Capsid protein	1,317	a→g	H106R	No
				F	Capsid protein	1,461	a→g	Q154R	No
				H	Minor spike protein	3,132	g→a	A68T	No
R25 T4	3	*rfaH*	R5, R18, R8, R10, R26, R12, R14, R25, R11, R7, R4, R13, R20, R21, R24, R29, R33, R2, R34, WT	*F*	Capsid protein	1,301	a→g	T101A	Yes (in C1 T21)
				F	Capsid protein	1,304	g→c	G102R	No
				F	Capsid protein	1,347	a→g	D116G	No
				H	Minor spike protein	3,132	g→a	A68T	No
R6 T12	3	*waaP*	R5, R18, R27, R31, R32, R6, R4, R13, R20, R21, R24, R29, R2, R34, WT	*F*	Capsid protein	1,727	c→t	L243F	No
				F	Capsid protein	2,015	g→c	D339H	No
				H	Minor spike protein	3,132	g→a	A68T	No
				H	Minor spike protein	3,154	c→t	A75V	No

Table S2: List of mutations identified in ΦX174 evolved isolates. ΦX174 strains: R# is the corresponding resistant strain number and T# is the transfer number where plaques were observed for the first time on the given resistant strain. Evolution experiment #: experiment number from which the evolved phages were isolated. Mutants sensitive to the evolved ΦX174 strains infection: list of all resistant strains that a specific phage strain can infect (See **Figs. 6 and S3**). Descriptions: names of proteins encoded by the listed genes. Positions: genomic coordinates of mutation (according to GenBank accession number AF176034.1). Nucleotide changes: observed nucleotide change. Amino acid changes: resulting change in amino acid sequence. Are mutations present in a control lineage?: indicates mutations that were observed in at least one control lineage (see **Methods**).

ΦX174 populations	Gene mutated in the corresponding *E. coli* C resistant strains	Gene mutated in ΦX174 strains	Descriptions	Positions	Nucleotide changes	Amino acid changes	Frequencies (%)	Are mutations present in a control lineage?
C1 T21	None (WT)	*F*	Capsid protein	1,301	a→g	T101A	69,8	-
		F	Capsid protein	1,965	g→a	G322D	70,9	-
C2 T21	None (WT)	*F*	Capsid protein	1,968	a→g	N323S	17,8	-
		F	Capsid protein	2,277	c→t	T426I	56,7	-
C3 T21	None (WT)	*F*	Capsid protein	2,277	c→t	T426I	5,7	-
		F	Capsid protein	2,280	c→t	S247L	80,7	-
R2 T21	*waaW*	*F*	Capsid protein	1,965	g→a	G322D	19,0	Yes (in C1 T21)
		F	Capsid protein	2,276	a→g	T426A	30,4	No
		F	Capsid protein	2,279	t→c	S427P	10,9	No
		F	Capsid protein	2,280	c→t	S247L	30,9	Yes (in C3 T21)
R5 T21	*gmhB*	NA	NA	NA	NA	NA	NA	NA
R7 T21	*waaO*	*F*	Capsid protein	2,279	t→c	S427P	5,4	
		F	Capsid protein	2,280	c→t	S247L	93,2	Yes (in C3 T21)
R9 T21	*waaG*	*F*	Capsid protein	2,280	c→t	S247L	100	Yes (in C3 T21)
R11 T21	*waaP*	NA	NA	NA	NA	NA	NA	NA
R12 T21	*waaF*	*F*	Capsid protein	2,280	c→t	S247L	100	Yes (in C3 T21)
R14 T21	*waaF*	*F*	Capsid protein	2,280	c→t	S247L	93,5	Yes (in C3 T21)
R16 T21	*galU*	*F*	Capsid protein	2,280	c→t	S247L	100	Yes (in C3 T21)
R17 T21	*galU*	*F*	Capsid protein	2,279	t→c	S427P	6,7	No
		F	Capsid protein	2,280	c→t	S247L	93,1	Yes (in C3 T21)

R23 T21	*waaG*	*F*	Capsid protein	1,301	a→g	T101A	5,6	Yes (in C1 T21)
		F	Capsid protein	2,280	c→t	S247L	91,6	Yes (in C3 T21)
R30 T21	*galU*	*F*	Capsid protein	2,279	t→c	S427P	7,9	No
		F	Capsid protein	2,280	c→t	S247L	90,0	Yes (in C3 T21)
R32 T21	*hldE*	*F*	Capsid protein	2,276	a→g	T426A	29,6	No
		F	Capsid protein	2,277	c→t	T426I	6,8	Yes (in C2 T21 and C3 T21)
		F	Capsid protein	2,280	c→t	S247L	61,9	Yes (in C3 T21)
R33 T21	*waaT*	*F*	Capsid protein	2,276	a→g	T426A	42,9	No
		F	Capsid protein	2,277	c→t	T426I	14,8	Yes (in C2 T21 and C3 T21)
		F	Capsid protein	2,280	c→t	S247L	41,7	Yes (in C3 T21)
		*A/A**	Replication-associated protein/protein A*	4,817	c→t	D279D/D107D	12,8	No
R34 T21	*waaW*	NA	NA	NA	NA	NA	NA	NA
R35 T21	*galU*	*F*	Capsid protein	1,301	a→g	T101A	19,1	Yes (in C1 T21)
			Capsid protein	1,649	c→t	R217C	8,0	No
			Capsid protein	2,179	g→t	Q393H	6,8	No
			Capsid protein	2,280	c→t	S247L	82,9	Yes (in C3 T21)

Table S3: List of mutations found in the evolved ΦX174 populations that did not infect their corresponding *E. coli* C resistant strains. ΦX174 populations: R# is the corresponding resistant strain number and T# is the transfer number where plaques were observed for the first time on the given resistant strain. Descriptions: names of proteins encoded by the listed genes. Positions: genomic coordinates of mutation (according to GenBank accession number AF176034.1). Nucleotide changes: observed nucleotide change. Amino acid changes:

resulting change in amino acid sequence. Frequencies (%): frequency of a given mutation in the phage population. Are mutations present in a control lineage?: indicates mutations that were observed in at least one control lineage (see **Methods**). NA: no data available.

Excluded *E. coli* C resistant strains	Genes	Locus tags	Synonyms	Descriptions	Positions	Nucleotide changes	Amino Acid changes	Predicted LPS structure phenotypes
R1	*waaV*	*B6N50_00435* →	-	UDP-glucose:(Glucosyl) LPS β1,3-Glucosyltransferase	80,534	$(t)_{7\rightarrow6}$	F149fsTer#158[a]	Rough
	galE	*B6N50_15815* →	-	UDP-glucose 4-epimerase	3,096,098 - 3,096,107	Δ10 bp	T228fsTer#233	
R3	*galE*	*B6N50_15815* →	-	UDP-glucose 4-epimerase	3,095,443	g→a	G10D	Rough
R15	*galE*	*B6N50_15815* →	-	UDP-glucose 4-epimerase	3,096,191	c→a	Y259stop	Rough
R19	*yajC*	*B6N50_17610* ←	-	Preprotein translocase subunit	3,447,574	Δ1 bp	G18fsTer#23	Wildtype

ΦX174 strains	Evolution experiment #	Gene mutated in the corresponding *E. coli* C resistant strains	Gene mutated in ΦX174 strains	Descriptions	Positions	Nucleotide changes	Amino acid changes	Are mutations present in a control lineage?
R19 T1	1	*yajC*	*H*	Minor spike protein	3,781	c→a	S284Y	No
R5 T2	1	*gmhB*	*H*	Minor spike protein	3,169	c→t	G80V	No

Table S4: List of all *E. coli* C and evolved ΦX174 isolates excluded from the analysis. *E. coli* C resistant strains: R# is the excluded resistant strain number. Genes: name of gene(s) in which mutations have been identified (as compared with wildtype *E. coli* C; doi: 10.5281/zenodo.6952399). Locus tags: identifier of each listed gene. Synonyms: alternative gene names. Descriptions: protein product encoded by each listed gene. Positions: genomic coordinates of mutation. Nucleotide changes: observed nucleotide change. Amino acid changes: resulting change in amino acid sequence. Predicted LPS structure phenotypes: based on predicted LPS structure (see **Fig. 2**). “stop”: stop codon. “a”: F149fsTer#158 indicates a frameshift (fs) leading to a premature codon stop (Ter); the position of the premature stop codon is in parentheses. ΦX174 strains: R# is the corresponding resistant strain number and T# is the transfer number where plaques were observed for the first time on the given resistant strain. Evolution experiment #: experiment number where the evolved phages were obtained.

Descriptions: names of proteins encoded by the listed genes. Positions: genomic coordinates of mutation (according to GenBank accession number AF176034.1). Nucleotide changes: observed nucleotide change. Amino acid changes: resulting change in amino acid sequence. Are mutations present in a control lineage?: indicates mutations that were observed in at least one control lineage (see **Methods**).

E. coli C resistant strains	Replicate #	Genes	Locus tags	Descriptions	Positions	Nucleotide changes	Amino Acid changes	Predicted LPS structure phenotypes
R3	1	*gltD*	*B6N50_02830* ←	Glutamate synthase subunit gltD	555,954	t→c	Y318C	Rough
		galE	*B6N50_15815* →	UDP-glucose 4-epimerase	3,095,443	g→a	G10D	
R3	2, 3, 5, 9, 10	*WaaT*	*B6N50_00420* →	UDP-galactose:(glucosyl) LPS α-1,2-galactosyltransferase	77,302	$(a)_{6\rightarrow5}$	K16fsTer#23[a]	Rough
		gltD	*B6N50_02830* ←	Glutamate synthase subunit gltD	555,954	t→c	Y318C	
		-	-	Hypothetical protein	2,147,627	c→t	Q793stop	
		galE	*B6N50_15815* →	UDP-glucose 4-epimerase	3,095,443	g→a	G10D	
R3	4	*oppB*	*B6N50_13280* ←	Murein tripeptide ABC transporter/inner membrane subunit	2,600,004	g→a	A211V	Deep rough
		galU	*B6N50_13325* ←	UTP--glucose-1-phosphate uridylyltransferase	2,611,933	Δ1 bp	I57fsTer#86	
		galE	*B6N50_15815* →	UDP-glucose 4-epimerase	3,095,443	g→a	G10D	
R3	6	*gmhD*	*B6N50_00455* ←	ADP-L-glycero-D-mannoheptose-6-epim erase	84,986	g→t	Y140stop	Rough
		galE	*B6N50_15815* →	UDP-glucose 4-epimerase	3,095,443	g→a	G10D	
R3	7	*galE*	*B6N50_15815* →	UDP-glucose 4-epimerase	3,095,443	g→a	G10D	Rough
		galT	*B6N50_15820* →	Galactose-1-phosphate uridylyltransferase	3,097,396	t→c	L319P	
R3	8	*gltD*	*B6N50_02830* ←	Glutamate synthase subunit gltD	555,954	t→c	Y318C	Rough
		-	-	Hypothetical protein	2,147,627	c→t	Q793stop	
		galE	*B6N50_15815* →	UDP-glucose 4-epimerase	3,095,443	g→a	G10D	
R15	1	*galP*	*B6N50_04295* ←	Galactose-proton symporter	835,258	a→g	F405L	Rough
		galE	*B6N50_15815* →	UDP-glucose 4-epimerase	3,096,191	c→a	Y259stop	

R15	2	*WaaT*	*B6N50_00420* →	UDP-galactose:(glucosyl) LPS α-1,2-galactosyltransferase	77,590	g→t	E112stop	Rough
		galE	*B6N50_15815* →	UDP-glucose 4-epimerase	3,096,191	c→a	Y259stop	
R15	3 - 6	*waaW*	*B6N50_00430* →	UDP-galactose--(galactosyl) LPS alpha1,2-galactosyltransferase	79,764	t→g	L262R	Rough
		galE	*B6N50_15815* →	UDP-glucose 4-epimerase	3,096,191	c→a	Y259stop	
R15	7	*galP*	*B6N50_04295* ←	Galactose-proton symporter	836,442	g→t	S10stop	Rough
		galE	*B6N50_15815* →	UDP-glucose 4-epimerase	3,096,191	c→a	Y259stop	
R15	8	*galP*	*B6N50_04295* ←	Galactose-proton symporter	835,353	a→g	L373P	Rough
		galE	*B6N50_15815* →	UDP-glucose 4-epimerase	3,096,191	c→a	Y259stop	
R15	9	*galE*	*B6N50_15815* →	UDP-glucose 4-epimerase	3,096,191	c→a	Y259stop	Rough
R15	10	*galP*	*B6N50_04295* ←	Galactose-proton symporter	835,170	c→t	W434stop	Rough
		galE	*B6N50_15815* →	UDP-glucose 4-epimerase	3,096,191	c→a	Y259stop	

Table S5: List of the additional mutations associated with *galE* mutations. *E. coli* C resistant strains: R# is the resistant strain number. Genes: name of the gene(s) in which mutations have been identified (as compared with wildtype *E. coli* C; doi: 10.5281/zenodo.6952399). Locus tags: identifier of each listed gene. Descriptions: protein product encoded by each listed gene. Positions: genomic coordinates of mutation. Nucleotide changes: observed nucleotide change. Amino acid changes: resulting change in amino acid sequence. Predicted LPS structure phenotypes: based on predicted LPS structure (see **Fig. 2**). “stop”: stop codon. “[a]”: K16sTer#23 indicates a frameshift (fs) leading to a premature codon stop (Ter); the position of the premature stop codon is in parentheses.

Name	Sequence (5’→3’)	Binding Region	Tm 2Q5	Ta 2Q5	Purpose
ΦX174_2361_r	TCGCTTGGTCAACCCCTCAG	2342-2361	70°C	71°C	ΦX174 Whole genome amplification
ΦX174_2362_f	AGCGCGGTAGGTTTTCTGCT	2362-2382	70°C		
ΦX174_399_r	CTTGACTCATGATTTCTTACC	379-399	58°C	59°C	ΦX174 Whole genome amplification
ΦX174_400_f	TTACTGAACAATCCGTACGTTTC	400-422	62°C		

Name	Sequence (5’→3’)	Binding Region	Tm Phusion (buffer HF)	Ta Phusion (buffer HF)	Purpose
ΦX174_F_fw	CGCTCGTCTTTGGTATGTAGGTGG	928-951	65°C	68°C	Amplification of ΦX174's *F* gene
ΦX174_F_rv	AGCGGCGTTGACAGATGTATCC	2580-2601	65°C		Amplification of ΦX174's *F* gene
ΦX174_F_fw2	GTTGCGAGGTACTAAAGGCAAGC G	898-921	66°C	68°C	Amplification of ΦX174's *F* gene
ΦX174_F_rv2	CTGGAGTAACAGAAGTGAGAACCA GC	2245-2470	65°C		Amplification of ΦX174's *F* gene
ΦX174_F_fw_sh	CGTCTTTGGTATGTAGGTGG	932-951	57°C	61°C	Amplification of ΦX174's *F* gene
ΦX174_F_rv_sh	CGGCGTTGACAGATGTATC	2581-2599	58°C		Amplification of ΦX174's *F* gene
ΦX174_H_fw	CAAGGACTGTGTGACTATTGACGT CC	2763-2788	65°C	68°C	Amplification of ΦX174's *H* gene
ΦX174_H_rv	GCCTCTACGCGATTTCATAGTGGA G	4490-4514	65°C		Amplification of ΦX174's *H* gene
ΦX174_H_fw2	CGCTACTAAATGCCGCGGATTGG	2847-2869	66°C	67°C	Amplification of ΦX174's *H* gene
ΦX174_H_rv2	GCCTCCAGCAATCTTGAACACTC	4467-4489	64°C		Amplification of ΦX174's *H* gene
ΦX174_H_fw_sh	GACTGTGTGACTATTGACGTCC	2767-2788	60°C	61°C	Amplification of ΦX174's *H* gene
ΦX174_H_rv_sh	GCCTCTACGCGATTTCATAG	4495-4514	58°C		Amplification of ΦX174's *H* gene

Name	Sequence (5’→3’)	Binding Region	Tm	Ta	Purpose
ΦX174_F_Sanger_fw	CCTCATCGTCACGTTTATGG	1217-1236	58°C	56°C	Sanger sequencing of ΦX174's *F* gene
ΦX174_F_Sanger_fw2	ACCGATATTGCTGGCGAC	1934-1951	59°C	56°C	Sanger sequencing of ΦX174's *F* gene
ΦX174_F_Sanger_rv	GGCGTTATAACCTCACACTC	2294-2313	58°C	56°C	Sanger sequencing of ΦX174's *F* gene
ΦX174_F_Sanger_rv2	GGCGAGAAAGCTCAGTCTC	1529-1547	60°C	56°C	Sanger sequencing of ΦX174's *F* gene
ΦX174_H_Sanger_fw	GTGGCGCCATGTCTAAATTG	2974-2993	59°C	56°C	Sanger sequencing of ΦX174's *H* gene
ΦX174_H_Sanger_fw2	CAGCAAACGCAGAATCAGC	3720-3738	60°C	56°C	Sanger sequencing of ΦX174's *H* gene
ΦX174_H_Sanger_rv	CCACAAGCCTCAATAGCAG	4054-4072	58°C	56°C	Sanger sequencing of ΦX174's *H* gene
ΦX174_H_Sanger_rv2	CTTTATCAGCGGCAGACTTG	3251-3270	58°C	56°C	Sanger sequencing of ΦX174's *H* gene

Table S6. List of primers used in this study. *F*: encodes for the viral capsid protein. *H*: encodes for the minor spike protein. “fw”: forward. “rv”: reverse**.** Tm 2Q5: melting temperature of the primer used with the Q5® High-Fidelity 2X Master Mix (NEB). Ta 2Q5: annealing temperature of the set of primers used with the Q5® High-Fidelity 2X Master Mix (NEB). Tm Phusion (buffer HF): melting temperature of the primer used with Phusion® High-Fidelity PCR Master Mix with HF Buffer (ThermoFisher). Ta Phusion (buffer HF): annealing temperature of the primer used with Phusion® High-Fidelity PCR Master Mix with HF Buffer (ThermoFisher).

Supplementary Methods

In this section, we provide a more detailed version of the protocols used in the **Methods** section.

First phage evolution experiment - Standard. The protocol from Bono *et al.* (Bono et al. 2012) was adapted to evolve phages that can infect resistant *E. coli* C strains. Of R1-R35 – the 35 *E. coli* C strains resistant to ΦX174 wildtype, see above – 31 were used for the first phage evolution experiment (excluding R1, R3, R15, and R19; see **Results** and **Text S1**). Thirty-one ΦX174 lineages were each founded by ΦX174 wildtype and serially transferred daily, for up to 21 days, on non-evolving host cultures containing a mix of (i) *E. coli* C wildtype (permissive strain) and (ii) one resistant *E. coli* C strain (non-permissive strain; R2-R35).

For each transfer, permissive and non-permissive bacteria were grown separately until they reached the log phase. These cultures were founded with an initial inoculum of 180 μl from fast and intermediate growers' overnight cultures or 350 μl from slow growers' overnight cultures (see **Fig. S4**). Fast and intermediate growers' cultures were incubated at 37˚C (250 rpm) for two hours, while slow growers' cultures were incubated for three hours. Then, permissive and non-permissive strains were mixed in a 14 ml sterile tube at a specific ratio depending on the growth of the non-permissive strain (1:1 for fast growers, 1:2 for intermediate growers, 1:3 for slow growers), to a final volume of 5 ml.

At each transfer, ~10^8 freshly prepared susceptible cells were infected by ~10^7 ΦX174 (MOI_{input} ~0.1). Infected cultures were shaken at 250 rpm, 37˚C for three hours. Phage lysates were isolated as described in **Phage lysate preparation**. Phage lysates from the previous day were used to inoculate the fresh bacterial mixes the next day. Transfers continued until phages were found to infect their corresponding resistant *E. coli* C strain, or until 21 transfers were reached. Three control lines were also run in parallel, by transferring ΦX174 at MOI_{input} ~0.1 in a culture containing *E. coli* C wildtype (permissive strain) only.

Second phage evolution experiment – Increased diversity. A phage cocktail made of (i) the wildtype ΦX174 and (ii) all ΦX174 strains that successfully re-infected their corresponding resistant strains during the phage evolution experiment – Standard was generated by first diluting each phage lysate and mixing them at a roughly equal number of pfu (final concentration of ~2.10^8 pfu ml^{-1}). These phage strains (14 in total) are: ΦX174 R4 T7, ΦX174 R5 T2, ΦX174 R8 T6,

ΦX174 R10 T6, ΦX174 R13 T6, ΦX174 R18 T4, ΦX174 R19 T1, ΦX174 R20 T5, ΦX174 R21 T1, ΦX174 R24 T1, ΦX174 R26 T1, ΦX174 R27 T1, ΦX174 R29 T1, and ΦX174 R31 T1. Despite being removed from the mutational analysis, phages infecting R5 and R19, respectively, were part of the final cocktail (see **Text S1** and **Table S4**).

The phage cocktail was grown and transferred daily, for up to four days, on non-evolving host cultures containing: (i) *E. coli* C wildtype, (ii) the 14 host strains for which resistance had been overcome (permissive hosts: *E. coli* C R4, R5, R8, R10, R13, R18, R19, R20, R21, R24, R26, R27, R29, and R31), and (iii) an excess of one of the still-resistant strain of interest (non-permissive host). For the non-permissive host, we used R6 (*waaP/pssA* mutant), R22 (*galU* mutant), R25 (*rfaH* mutant), or R28 (*waaG* mutant; see **Table S1**). *E. coli* C R22 and R28 were chosen as representatives of the *galU* and *waaG* mutants, respectively.

Each bacterial strain (permissive and non-permissive) was grown separately in LB liquid culture for one hour (37˚C, 250 rpm). An inoculum of 180 µl from overnight cultures was used for the fast and intermediate growers, while 350 µl was used for the slow growers. After the incubation time, permissive strains were pooled at a roughly equal number of colony-forming units (CFU), for a final concentration of ~10^8 susceptible, permissive cells. One of the *E. coli* C resistant strains of interest (non-permissive host; R6, R22, R25 or R28) was then added in excess (in between ~1 and $5x10^8$ resistant cells) in the host mix, to give a final volume of 4 ml.

Infections were initiated by adding ~10^7 phages (MOI_{input} ~0.1) from the phage cocktail to the bacterial culture mix. Infected cultures were shaken in 100 ml sterile Erlenmeyer at 250 rpm, 37˚C, for five hours. Phage lysates were isolated as described in **Phage lysate preparation**. Phage lysates from the previous day were used to inoculate the fresh bacterial mixes the next day. This process was repeated until phages were found to infect their corresponding resistant *E. coli* C strains, or until four transfers had been completed.

Third evolution experiment – Increased diversity and generations. The same phage cocktail used in the second phage evolution experiment – Increased diversity was grown and transferred daily, four times a day, for up to four days on non-evolving host cultures containing (i) *E. coli* C wildtype, (ii) the 14 host strains for which resistance had been overcome (permissive hosts: *E. coli* C R4, R5, R8, R10, R13, R18, R19, R20, R21, R24, R26, R27, R29, and R31), and (iii) one of the still-resistant strain of interest (non-permissive host). The definitive protocol from the second

phage evolution experiment – Increased diversity was adjusted to reduce the amount of time necessary to retrieve the desired phages. We increased the number of transfers per day from one to four for a total of four days (16 transfers in total). Only *E. coli* C R6 (*waaP*/*pssA* mutant) and R25 (*rfaH* mutant) (see **Table S1**) were used as non-permissive hosts.

Each bacterial strain (permissive and non-permissive) was grown separately for one hour. An inoculum of 180 μl from overnight cultures was used for the fast and intermediate growers, while 350 μl was used for the slow growers. After the incubation time, permissive strains were pooled at a roughly equal number of colony-forming units (CFU) in three different mixes depending on the growth categories of each bacterium (slow, intermediate, and fast growers, **Fig. S4**). 1 ml of each fast grower was used to make the "fast mix" (constituted of *E. coli* C wildtype, R5, R19, R27, and R31). 833 μl of each intermediate grower was used for the "intermediate mix" (constituted of R4, R13, R20, R21, R24, and R29). Finally, 1.25 ml of each slow grower was used to prepare the "slow mix" (constituted of R8, R10, R18, and R26).

Non-permissive strains and bacterial mixes were kept in exponential growth phase independently by transferring 1:5 of the volume (1 ml) in fresh LB pre-heated at 37˚C every hour (final volume of 5 ml). To check whether the growth of the different bacteria remained consistent from one transfer to another, the OD600 values were measured before each transfer. The volume transferred at each hour was modified accordingly if the bacterial growth was too fast or too slow.

Before adding the phage cocktail, all bacterial mixes were pooled together at a roughly equal number of cfu (500 μl each, for a total of $\sim 10^{8}$ susceptible cells), and one of the *E. coli* C resistant strains of interest (non-permissive host; R6 or R25) was added in excess (i.e., the number of resistant cells was comprised between ~1 and 5.10^{8}), for a final volume of 4 ml.

Infections started by adding $\sim 10^{7}$ phages (MOI_{input} ~0.1) from the cocktail in the final bacterial mix. Infected cultures were shaken in 100 ml sterile Erlenmeyer at 250 rpm, 37˚C, for one hour. After that, 1:15 of the volume of each infected culture was transferred to freshly mixed bacterial cultures and the infection continued for one hour. This step was repeated for a total of four transfers. The final (fourth) transfer lasted for two hours to ensure that all phages completed the infection cycle and are present in the culture medium and not in the cell. All transfers completed on the same day involved transferring both phage and bacteria; on every fourth transfer, supernatants were collected and only phages were transferred. Phage lysates were isolated as

described in **Phage lysate preparation**. Phage lysates from the previous day were used to inoculate the first fresh bacterial mixes the next day. This entire process was repeated until phages that could infect their corresponding resistant *E. coli* C strains were identified or until four transfers had been completed.

Isolation of evolved ΦX174 strains from single plaques. To isolate pure, single clones from the different evolution experiments, top agar overlays were prepared by mixing 100 μl of undiluted phage lysates with 200 μl of an overnight culture of the corresponding resistant *E. coli* C strain in 4 ml SSA (supplemented with $CaCl_2$ and $MgCl_2$ at a final concentration of 5 and 10 mM, respectively). Top agar overlays were poured onto LB plates, dried for at least 15 minutes, and incubated inverted at 37˚C for ~16-17 hours. Plaques were counted the next day. An isolated plaque was chosen randomly for each phage lysate to infect cultures of the corresponding resistant *E. coli* C strains (in exponential growth state) incubated at 37˚C, 250 rpm, for five hours. Phage lysates were isolated as described in **Phage lysate preparation**. To obtain pure phage glycerol stocks (isogenic stocks which are presumed to hold one genetic clone (van Charante et al. 2019)), phages were purified via a second round of plaque isolation from their first glycerol stocks. Phage lysates were isolated and stored as described in **Phage lysate preparation**.

Determination of the evolved phages' host range by spotting assays

Method 1. Each top agar overlay was prepared by mixing 200 μl of an *E. coli* C strain from stationary phase culture in 4 ml SSA (supplemented with $CaCl_2$ and $MgCl_2$ at a final concentration of 5 and 10 mM, respectively), then poured on LB plates and dried for at least 15 minutes under a sterile laminar flow hood. Then, 3 μl of each undiluted evolved phage lysate (between 10^7 and 10^9 pfu ml^{-1}) was dropped at the surface.

Method 2. Each top agar overlay was prepared by mixing a volume of each phage lysate in 4 ml SSA at a final concentration of ~10^7 pfu ml^{-1}, then poured on LB plates and dried for at least 15 minutes under a sterile laminar flow hood. Then, 3 μl of both undiluted and ten-fold diluted of each *E. coli* C strain (from overnight culture) were dropped onto the surface with a pipette.

For both methods, spots were dried for at least 30 minutes under sterile laminar flow, and the plates were subsequently incubated inverted at 37˚C for ~17 hours. *E. coli* C wildtype was used as a positive control (permissive strain), and *E. coli* K-12 MG1655 as a negative control (non-permissive strain). Sterile H_2O was also spotted at the end of each plate as a control for material

contamination (pipettes and tips). A bacterial host strain was classified as sensitive only when signs of lysis were detected using both methods, in at least two (of three) replicates per method. A phage-bacterium combination that yielded different results between the two methods was tested in *standard plaque assays* as follows: a top agar overlay was prepared by mixing 100 μl of the phage lysate diluted to 10^{-1} and 10^{-6} with 200 μl of the resistant *E. coli* C strain from stationary phase culture in 4 ml SSA supplemented with $CaCl_2$ and $MgCl_2$ at a final concentration of 5 and 10 mM, respectively), then poured on LB plates and dried for at least 15 minutes. The presence or absence of plaques was assessed the next day. Finally, the host strain was classified as sensitive if plaques were observed at both dilutions.

References

Amor K, Heinrichs DE, Frirdich E, Ziebell K, Johnson RP, Whitfield C. 2000. Distribution of core oligosaccharide types in lipopolysaccharides from *Escherichia coli*. *Infect Immun*. 68:1116–1124.

Belunis CJ, Clementz T, Carty SM, Raetz CRH. 1995. Inhibition of lipopolysaccharide biosynthesis and cell growth following inactivation of the *kdtA* gene in *Escherichia coli*. *J Biol Chem*. 270:27646–27652.

Bohm K, Porwollik S, Chu W, Dover JA, Gilcrease EB, Casjens SR, McClelland M, Parent KN. 2018. Genes affecting progression of bacteriophage P22 infection in *Salmonella* identified by transposon and single gene deletion screens: host genes affecting phage P22 infection. *Mol Microbiol*. 108:288–305.

Bono LM, Gensel CL, Pfennig DW, Burch CL. 2012. Competition and the origins of novelty: experimental evolution of niche-width expansion in a virus. *Biol Lett*. 9:20120616–20120616.

van Charante F, Holtappels D, Blasdel B, Burrowes B. 2019. Isolation of bacteriophages. In: Harper DR, Abedon ST, Burrowes BH, McConville ML, editors. Bacteriophages: Biology, Technology, Therapy. Springer, Cham. p. 1–32.

Fang J, Wei Y. 2011. Expression, purification and characterization of the *Escherichia coli* integral membrane protein YajC. *Protein Pept Lett*. 18:601–608.

Frey PA. 1996. The Leloir pathway: a mechanistic imperative for three enzymes to change the stereochemical configuration of a single carbon in galactose. *FASEB j*. 10:461–470.

Genevaux P, Bauda P, DuBow MS, Oudega B. 1999. Identification of Tn10 insertions in the *rfaG*, *rfaP*, and *galU* genes involved in lipopolysaccharide core biosynthesis that affect *Escherichia coli* adhesion. *Arch of Microbiol*. 172:1–8.

Hancock RE, Reeves P. 1976. Lipopolysaccharide-deficient, bacteriophage-resistant mutants of *Escherichia coli* K-12. *J Bacteriol*. 127:98–108.

Heinrichs DE, Yethon JA, Amor PA, Whitfield C. 1998. The assembly system for the outer core portion of R1- and R4-type lipopolysaccharides of *Escherichia coli*: the R1 core-specific β-glucosyltransferase provides a novel attachment site for O-polysaccharides. *J Biol Chem*. 273:29497–29505.

Heinrichs DE, Yethon JA, Whitfield C. 1998. Molecular basis for structural diversity in the core regions of the lipopolysaccharides of *Escherichia coli* and *Salmonella enterica*. *Mol Microbiol*. 30:221–232.

Jansson P-E, Lindberg B, Lindberg AA, Wollin R. 1981. Structural studies on the hexose region of the core in lipopolysaccharides from Enterobacteriaceae. *Eur J Biochem*. 115:571–577.

Karp PD, Billington R, Caspi R, Fulcher CA, Latendresse M, Kothari A, Keseler IM, Krummenacker M, Midford PE, Ong Q, et al. 2019. The BioCyc collection of microbial genomes and metabolic pathways. *Brief Bioinform*. 20:1085–1093.

Kawaura T, Inagaki M, Karita S, Kato M, Nishikawa S, Kashimura N. 2000. Recognition of receptor lipopolysaccharides by spike G protein of bacteriophage ΦX174. *Biosci Biotechnol Biochem*. 64:1993–1997.

Klein G, Lindner B, Brabetz W, Brade H, Raina S. 2009. *Escherichia coli* K-12 suppressor-free mutants lacking early glycosyltransferases and late acyltransferases: minimal

lipopolysaccharide structure and induction of envelope stress response. *J Biol Chem.* 284:15369–15389.

Klein G, Müller-Loennies S, Lindner B, Kobylak N, Brade H, Raina S. 2013. Molecular and structural basis of inner core lipopolysaccharide alterations in *Escherichia coli*: incorporation of glucuronic acid and phosphoethanolamine in the heptose region. *J Biol Chem.* 288:8111–8127.

Kneidinger B, Marolda C, Graninger M, Zamyatina A, McArthur F, Kosma P, Valvano MA, Messner P. 2002. Biosynthesis pathway of ADP-L-glycero-β-D-manno-heptose in *Escherichia coli. J. Bacteriol.* 184:363-369.

Król JE, Hall DC, Balashov S, Pastor S, Sibert J, McCaffrey J, Lang S, Ehrlich RL, Earl J, Mell JC, et al. 2019. Genome rearrangements induce biofilm formation in *Escherichia coli* C – an old model organism with a new application in biofilm research. *BMC Genomics* 20:767.

Kulikov EE, Golomidova AK, Prokhorov NS, Ivanov PA, Letarov AV. 2019. High-throughput LPS profiling as a tool for revealing of bacteriophage infection strategies. *Sci Rep.* 9:2958.

Labrie SJ, Samson JE, Moineau S. 2010. Bacteriophage resistance mechanisms. *Nat Rev Microbiol.* 8:317–327.

Leipold MD, Vinogradov E, Whitfield C. 2007. Glycosyltransferases involved in biosynthesis of the outer core region of *Escherichia coli* lipopolysaccharides exhibit broader substrate specificities than is predicted from lipopolysaccharide structures. *J Biol Chem.* 282:26786–26792.

van der Ley P, de Graaff P, Tommassen J. 1986. Shielding of *Escherichia coli* outer membrane proteins as receptors for bacteriophages and colicins by O-antigenic chains of lipopolysaccharide. *J Bacteriol* 168:449–451.

Matsuura M. 2013. Structural modifications of bacterial lipopolysaccharide that facilitate Gram-Negative bacteria evasion of host innate immunity. *Front Immunol.* 109:4.

McArthur F, Andersson CE, Loutet S, Mowbray SL, Valvano MA. 2005. Functional analysis of the glycero-manno-heptose 7-phosphate kinase domain from the bifunctional HldE protein, which is involved in ADP-L-glycero-D-manno-heptose biosynthesis. *J Bacteriol.* 187:5292–5300.

Mutalik VK, Adler BA, Rishi HS, Piya D, Zhong C, Koskella B, Kutter EM, Calendar R, Novichkov PS, Price MN, et al. 2020. High-throughput mapping of the phage resistance landscape in *E. coli*. *PLoS Biol.* 18:e3000877.

Pagnout C, Sohm B, Razafitianamaharavo A, Caillet C, Offroy M, Leduc M, Gendre H, Jomini S, Beaussart A, Bauda P, et al. 2019. Pleiotropic effects of *rfa*-gene mutations on *Escherichia coli* envelope properties. *Sci Rep.* 9:9696.

Pierson DE, Carlson S. 1996. Identification of the *galE* gene and a *galE* homolog and characterization of their roles in the biosynthesis of lipopolysaccharide in a serotype O:8 strain of *Yersinia enterocolitica*. *J Bacteriol.* 178:5916–5924.

Raetz CRH, Whitfield C. 2002. Lipopolysaccharide endotoxins. *Annu Rev Biochem.* 71:635–700.

Schnaitman CA, Austin EA. 1990. Efficient incorporation of galactose into lipopolysaccharide by *Escherichia coli* K-12 strains with polar *galE* mutations. *J Bacteriol.* 172:5511–5513.

Schnaitman CA, Klena JD. 1993. Genetics of lipopolysaccharide biosynthesis in enteric bacteria. *Microbiol Rev.* 57:655–682.

Schulze RJ, Komar J, Botte M, Allen WJ, Whitehouse S, Gold VAM, Nijeholt JAL a, Huard K, Berger I, Schaffitzel C, et al. 2014. Membrane protein insertion and proton-motive-force-dependent secretion through the bacterial holo-translocon SecYEG–SecDF–YajC–YidC. *PNAS* 111:4844–4849.

Vinogradov EV, van der Drift K, Thomas-Oates JE, Meshkov S, Brade H, Holst O. 1999. The structures of the carbohydrate backbones of the lipopolysaccharides from *Escherichia coli* rough mutants F470 (R1 core type) and F576 (R2 core type): LPS from *E. coli* R1 and R2 core types. *Eur J Biochem.* 261:629–639.

Weissborn AC, Liu Q, Rumley MK, Kennedy EP. 1994. UTP:α-D-glucose-L-phosphate uridylyltransferase of *Escherichia coli*: isolation and DNA Sequence of the *galU* gene and purification of the enzyme. *J Bacteriol.* 176:2611-2618.

Whitfield C, Heinrichs DE, Yethon JA, Amor KL, Monteiro MA, Perry MB. 1999. Assembly of the R1-type core oligosaccharide of *Escherichia coli* lipopolysaccharide. *J Endotoxin Res.* 5:151–156.

Yethon JA, Heinrichs DE, Monteiro MA, Perry MB, Whitfield C. 1998. Involvement of *waaY*, *waaQ*, and *waaP* in the modification of *Escherichia coli* lipopolysaccharide and their role in the formation of a stable outer membrane. *J Biol Chem.* 273:26310–26316.

Yethon JA, Vinogradov E, Perry MB, Whitfield C. 2000. Mutation of the lipopolysaccharide core glycosyltransferase encoded by *waaG* destabilizes the outer membrane of *Escherichia coli* by interfering with core phosphorylation. *J Bacteriol.* 182:5620–5623.
